# Blood T cell phenotypes correlate with fatigue severity in post-acute sequelae of COVID-19

**DOI:** 10.1007/s15010-023-02114-8

**Published:** 2023-11-04

**Authors:** Isabell Pink, Jan K. Hennigs, Louisa Ruhl, Andrea Sauer, Lennart Boblitz, Marie Huwe, Jan Fuge, Christine S. Falk, Thomas Pietschmann, Martina de Zwaan, Antje Prasse, Stefan Kluge, Hans Klose, Marius M. Hoeper, Tobias Welte

**Affiliations:** 1 Department of Respiratory Medicine and Infectious Diseases, Hannover Medical School, Carl-Neuberg-Str. 1, 30625 Hannover, Germany; 2 Biomedical Research in Endstage and Obstructive Lung Disease Hannover (BREATH), German Center for Lung Research (DZL), Hannover, Germany; 3 Division of Respiratory Medicine, II. Department of Medicine, University Medical Center Hamburg-Eppendorf, 20251 Hamburg, Germany; 4 Institute of Transplant Immunology, Hannover Medical School, Hannover, Germany; 5 German Center for Infection Research (DZIF), TTU-IICH, Hannover, Germany; 6 Institute of Experimental Virology, Hannover Medical School, TWINCORE Research Center, Hannover, Germany; 7 Department of Psychosomatic Medicine and Psychotherapy, Hannover Medical School, Hannover, Germany; 8 Department of Intensive Care Medicine, University Medical Center Hamburg-Eppendorf, 20251 Hamburg, Germany

**Keywords:** Long-COVID, SARS-CoV-2, Neutralizing antibodies, Immune phenotypes, Fatigue assessment scale, Immunodysregulation

## Abstract

**Purpose:**

Post-acute sequelae of COVID-19 (PASC) affect approximately 10% of convalescent patients. The spectrum of symptoms is broad and heterogeneous with fatigue being the most often reported sequela. Easily accessible blood biomarkers to determine PASC severity are lacking. Thus, our study aimed to correlate immune phenotypes with PASC across the severity spectrum of COVID-19.

**Methods:**

A total of 176 originally immunonaïve, convalescent COVID-19 patients from a prospective cohort during the first pandemic phase were stratified by initial disease severity and underwent clinical, psychosocial, and immune phenotyping around 10 weeks after first COVID-19 symptoms. COVID-19-associated fatigue dynamics were assessed and related to clinical and immune phenotypes.

**Results:**

Fatigue and severe fatigue were commonly reported irrespective of initial COVID-19 severity or organ-specific PASC. A clinically relevant increase in fatigue severity after COVID-19 was detected in all groups. Neutralizing antibody titers were higher in patients with severe acute disease, but no association was found between antibody titers and PASC. While absolute peripheral blood immune cell counts in originally immunonaïve PASC patients did not differ from unexposed controls, peripheral CD3^+^CD4^+^ T cell counts were independently correlated with fatigue severity across all strata in multivariable analysis.

**Conclusions:**

Patients were at similar risk of self-reported PASC irrespective of initial disease severity. The independent correlation between fatigue severity and blood T cell phenotypes indicates a possible role of CD4^+^ T cells in the pathogenesis of post-COVID-19 fatigue, which might serve as a blood biomarker.

**Supplementary Information:**

The online version contains supplementary material available at 10.1007/s15010-023-02114-8.

## Introduction

In an enormous global endeavor, > 300,000 clinical and experimental studies related to COVID-19 have been published, addressing topics from infection dynamics to potential long-term consequences and sequelae of COVID-19 (as of early 2023).

On average, 81% of patients infected with the severe acute respiratory syndrome corona virus 2 (SARS-CoV-2) had a mild disease course during the initial phase of the pandemic with the wildtype virus (D614G variant). Around 14% exhibited respiratory complications including tachypnea, radiological lung infiltrates, or need for oxygen supplementation, while five percent of the patients were critically ill due to severe respiratory failure, hyperinflammation, and other organ dysfunctions [1]. The early pandemic phase saw an overall mortality rate of approx. 2.3%, which was elevated to 50% in high-risk patients with severe COVID-19 [1, 2]. With the introduction of specific antiviral and anti-inflammatory treatments, along with evolving virus strains and widespread vaccination, disease severity and mortality rates have decreased [3].

Beyond the acute disease phase, both physical and mental long-term consequences of COVID-19 have been reported very early on. The World Health Organization (WHO) estimates that 10–20% of those infected may experience post-acute COVID-19 sequela (PACS) [4] spanning multiple organ systems [5]. Identified risk factors for PACS include female sex, more severe acute disease, and a higher number of symptoms during COVID-19 [6–8]. Most commonly reported symptoms include cardiopulmonary sequelae such as exertional dyspnea and tachycardia as well as reduced quality of life, anxiety and, most frequently, fatigue [9]. While the humoral immune response, specifically the presence of specific immunoglobulin G (IgG) antibodies against the spike protein S1, was linked with an increased risk of PASC [10], the role of dysregulated cellular immunity became a later focus of research [11]. Despite the extensive research into PACS, there remains a notable absence of reliable blood-based biomarkers specifically for fatigue.

In the present study, we therefore conducted a prospective analysis on 176 previously immunonaïve, convalescent COVID-19 patients from the first pandemic phase with the D614G wildtype virus from two large university hospitals in Northern Germany (UKE, Hamburg and MHH, Hannover). We investigated the associations between common COVID-19 sequelae, emphasizing particularly on fatigue, and various peripheral blood immune phenotypes to identify possible biomarkers of PASC. This included a detailed exploration of cellular immunity patterns and B cell responses across three patient cohorts: non-hospitalized, hospitalized (non-ICU), and hospitalized (ICU).

## Methods

### Study design and patient characteristics

Patients following an infection with the D614G wildtype virus were recruited via prospective follow-up programs started in April 2020 or specialized outpatient clinics of the Departments of Respiratory Medicine by Hannover Medical School (MHH, 124 patients) and the University Medical Center Hamburg-Eppendorf (UKE, 52 patients). Adult patients (≥ 18 years) with proven previous SARS-CoV-2 infection detected by virus-specific RT-qPCR from nasopharyngeal swabs (cycle threshold values < 34) were included in this study. Baseline characteristics are shown in Table 1. An additional control cohort of 33 unexposed and unvaccinated individuals (19 females [58%], 14 males [42%], mean age 45·6 years) was recruited by an open call between May and September 2020 through the Institute of Transplantation Immunology of the MHH. Written informed consent was obtained from each patient.

**Table 1:** Baseline characteristics of non-hospitalized, hospitalized non-ICU, and ICU patients

	All *n* = 176	Non-hospitalized patients *n* = 78	Hospitalized non-ICU patients *n* = 50	Hospitalized ICU patients *n* = 48	*p*-value
Site –* n* (%)					
Hannover MHH	124 (70.5%)	56 (45.2%)	32 (25.8%)	36 (29.0%)	0.462
Hamburg UKE	52 (29.5%)	22 (42.3%)	18 (34.6%)	12 (23.1%)	
Age – Median (IQR)	49.8 (38.7–58.9)	45.0 (33.2–51.7)	56.0 (48.2–65.4)	54.1 (42.0–63.3)	** < 0.001**
Sex – *n* (%)					
Female	79 (44·9%)	47 (60.3%)	18 (36.0%)	14 (29.2%)	
Male	97 (55.1%)	31 (39.7%)	32 (64.0%	34 (70.8%)	
Ethnic group					0.404
Caucasian	172 (97.7)	77 (98.7%)	49 (98.0%)	46 (95.8%)	
Asian	2 (1.1%)	1 (1.3%)	0 (0.0%)	1 (2.1%)	
African	1 (0.5%)	0 (0.0%)	0 (0.0%)	1 (2.1%)	
Hispanic	1 (0.5%)	0 (0.0%)	1 (2.0%)	0 (0.0%)	
Comorbidities – *n* (%)					
Diabetes	21 (11.9%)	1 (1.3%)	6 (12.0%)	14 (29.2%)	** < 0.001**
COPD	18 (10.2%)	5 (6.4%)	3 (6.0%)	10 (20.8%)	**0.017**
Cardiovascular diseases	23 (13.1%)	2 (2.6%)	10 (20.0%)	11 (22.9%)	**0.001**
Hypertension	44 (25.0%)	7 (9.0%)	16 (32.0%)	21 (43.8%)	** < 0.001**
Chronic renal failure	5 (2.8%)	0 (0%)	2 (4.0%)	3 (6.3%)	0.103
Obesity (BMI ≥ 30 kg/m^2^)	42 (23.9%)	10 (12.8%)	13 (26.0%)	19 (39.6%)	**0.003**
Liver disease	3 (1.7%)	0 (0%)	1 (2.0%)	2 (4.2%)	0.211
Depression	9 (5.1%)	1 (1.3%)	3 (6.0%)	5 (10.4%)	0.073
Covid-19-related initial symptoms – *n* (%)					
Loss of smell	86 (48.9%)	57 (73.1%)	17 (34.0%)	12 (25.0%)	** < 0.001**
Dyspnea	123 (69.9%)	41 (52.6%)	40 (80.0%)	42 (87.5%)	** < 0.001**
Dry Cough	108 (61.4%)	44 (56.4%)	32 (64.0%)	32 (66.7%)	0.467
Fever	132 (75.0%)	45 (57.7%)	44 (88.0%)	43 (89.6%)	** < 0.001**
Chest tightness	77 (43.8%)	37 (47.4%)	25 (50.0%)	15 (31.3%)	0.118
Fatigue	144 (81.8%)	70 (89.7%)	37 (74.0%)	37 (77.1%)	**0.048**
Headache	92 (52.3%)	49 (62.8%)	20 (40.0%)	23 (47.9%)	**0.032**
Diarrhea	38 (21.6%)	16 (20.5%)	10 (20.0%)	12 (25%)	0.795
WHO Improvement Scale (Disease Onset) – Median (IQR)	4 (2–5)	2 (2)	4 (3–4)	6 (5–7)	< 0.001**
WHO Improvement Scale (Visit) – Median (IQR)	2 (2–2)	2 (2)	2 (1–2)	2 (2)	0.066**
Days from symptom onset to visit		63 (51.8–99·5)	69.0 (60.3–87·3)	81.0 (65.5–110.0)	0.123**
Days from Symptoms to Hospitalization – Median (IQR)	–	–	7 (4.25–10.0)	7 (3.0–10.0)	
Mechanical Ventilation – *n* (%) – ICU only	–	–		30 (17% of all 61.2% of ICU)	
ECMO – *n* (%) – ICU Only	–	–	–	8 (4.5% of all·16.3% of ICU)	

*Mann–Whitney U-test, **ANOVA

*ICU* intensive care unit, *IQR* interquartile range, *COPD* chronic obstructive pulmonary disease, *BMI* body mass index, *WHO*: world health organization, *ECMO* extra-corporal membrane oxygenation

### Clinical phenotyping

The following assessment was performed in all patients: Pulmonary function testing (PFT) including body plethysmography, measurements of diffusion capacity for carbon monoxide (DLCO), arterialized capillary blood gas analysis (CBG) at rest, and assessment of the 6-min walking distance (6MWD, expressed as an absolute value and adjusted for age, sex, height, and weight (= % of predicted, %/pre)) with continuous measurement of peripheral oxygen saturation (SpO2), heart rate by pulse oximetry, and routine blood lab parameters (Table 2).

**Table 2:** Symptom burden and clinical impairment at follow-up

	All *n* = 176	Non-hospitalized patients *n* = 78	Hospitalized non-ICU patients *n* = 50	Hospitalized ICU patients *n* = 48	*p*-value
Days after symptom onset to first visit – median (IQR)	69.5 (58.0–101·0)	63.0 (51.8–99·5)	69 (60.3–87.3)	81.0 (65·5–110·0)	0.123**
Anthropometry – median (IQR)					
BMI in kg/cm^2^	26.7 (23.5–29.8)	24.6 (21.6–28.1)	27.8 (24·4–30.8)	28·4 (24·9–32.2)	< 0.001 **
Lung function –median (IQR)					
FVC %/predicted	97.0 (81.5–107.8)	102.0 (94.5–111.5)	94.5 (85.3–108.3)	80.0 (65.5–102.0)	< 0.001**
FEV_1_%/predicted	95.0 (83.0–105.0)	98.0 (89.0–106.5)	99.5 (86.0–107.5)	83.0 (71.0–99.5)	< 0.001**
FVC/FEV_1_%/predicted	85.0 (78.0–96.0)	82·0 (76·0–92.0)	85.0 (78.0–97.3)	88·0 (81·5–97·5)	0.037**
DLCO %/predicted	86.0 (71.0–98.5)	91.0 (83.5–102.0)	84.0 (68.3–102.0)	67.0 (50.5–83.5)	< 0.001**
TLC %/predicted	96.0 (84.0–107.0)	103.0 (94.5–110.0)	94.5 (84.0–105.0)	86.0 (73.0–98.5)	< 0.001**
PEF %/predicted	88.0 (75.3–103.8)	91.0 (79.5–104.0)	87.5 (78.5–103.0)	85.0 (70.5–104.0)	0.464
Capillary blood gases – median (IQR)					
pO_2_ in mm Hg	85.0 (78.7–91.3)	89.2 (83.5–93.2)	89.1 (83.9–95.7)	79.0 (76.2–85)	**0·011**
pCO_2_ in mm Hg	38.7 (36.0–41.0)	37.1 (34.2–39.9)	37.2 (35.2–40.3)	39.5 (35.6–41.2)	0.510
pH	7.42 (7.40–7.44)	7.43 (7.41–7.45)	7.43 (7.41–7.45)	7.42 (7.40–7.44)	0.282
sO_2_ in %	97.2 (96.4–98.0)	97.7 (96.8–98.4)	97.4 (96.5–98.1)	96.4 (95.2–97.0)	**0.014**
Six-minute walking test (6MWT) – median (IQR)					
Distance in m (6MWD)	561 (482–615)	585 (544–648)	550 (452–610)	483 (410–654)	< 0.001**
6MWD%/predicted	91 (82–102)	93 (84–103)	92 (82–110)	84 (74–94)	0.011**
SpO_2_ (delta)	− 1 (− 3–0)	0.0 (− 2.0–1.0)	– 1.0 (− 3.0–0.8)	− 2.0 (− 5.0–0.0)	0.053
Pulse pre/post in bpm	83/113 (73/98–96/126)	83/114 (74/101–94/129)	84/110 (70/98–97/126)	83/112 (69/97–98/123)	0.958/0·114
Pulse (delta)	31 (19–39)	31.0 (22.0–45.0)	30.5 (19.3–37.8)	27.0 (13.0–36.0)	0.069
- BORG-Scale pre/post	0/3 (0/1–2/4)	0/3 (0/1–2/4)	0/2 (0/0–2/3)	0/3 (0/1–2/4)	0.730/0.125
BORG-Scale (delta)	2 (0–3)	2.0 (0.5–3.0)	1.0 (0.0–2.0)	2.0 (0.0–3.0	0.081
Blood markers					
CRP in mg/l (*n* = 141)	2.0 (1·1–2.7)	2.0 (0.8–2.0)	2.0 (1.7–3.9)	2.0 (0.9–3.0)	0.110**
NT-proBNP in ng/l (*n* = 57)	74 (34–140)	49.5 (34.0–95.8)	57.0 (34·0–108.8)	205.0 (87.0–1064.0)	0.005**
Creatinine in µmol/l (*n* = 142)	76 (66–86)	75.0 (66.8–82.7)	77.9 (67.6–87.3)	74.0 (63.0–84.1)	0.332**
AST in U/l (*n* = 141)	23 (18–29)	21.0 (17.0–26.0)	26.0 (19.3–31.5)	22.0 (18.0–32.0	0.113**
ALT in U/l (*n* = 142)	24 (18–37)	20.0 (16.8–29.3)	26.0 (20.0–39.5)	26.0 (18.0–44.0)	0.157**
Post-COVID symptoms – *n* (%)					
Chest tightness	46 (26.1%)	26 (33.3%)	12 (24.0%)	8 (16.7%)	0.109
Cough	46 (26.1%)	25 (32.1%)	7 (14.0%)	14 (29.2%)	0.065
Fever	7 (4.0%)	2 (0.62%)	3 (6·0%)	2 (4.2%)	0.622
Headache	52 (29.5%)	28 (35.9%)	11 (22.0%)	13 (27.1%)	0.221
Quality of life and subjective condition					
Fatigue (Median) FAS ≥ 22 n (%), before disease onset	21 (11.9)	7 (9.0%)	8 (18.2%)	6 (12.8%)	0.510
FAS ≥ 35 n (%), before disease onset	1 (0.6)	1 (1.3%)	0 (0.0%)	0 (0.0%)	
FAS ≥ 22 n (%), after acute disease (first visit to clinic)	73 (41.5%)	35 (45.5%)	18 (40.9%)	20 (43.5)	0.412
FAS ≥ 35 n (%), after acute disease (first visit to clinic)	33 (18.8%)	19 (24.7%)	8 (18.2%)	6 (13.0%)	
FAS – Median (IQR)					
Before disease onset	15 (13–19)	15.0 (13.8–19.0)	15.0 (13.0–19.0)	15.0 (11.0–19.0)	0.599**
After acute disease o (1st visit to clinic)	25 (18–33)	28.0 (19.0–34.5)	23.0 (18.0–31.0)	24.0 (18.0–30.0)	0.151**
Difference (delta)	8 (2–17)	12.0 (2.5–18.0)	6.5 (2.0–15.0)	6.5 (2.8–15.0)	0.236**
Depression – *n* (%)					
None to minor	132 (78.1%)	61 (79.2%)	36 (80.0%)	35 (74.5%	0.638
Moderate to severe	37 (21.9%)	16 (20.8%)	9 (20.0%)	12 (25.6%)	
Anxiety – *n* (%)					
None to minor	123 (72·8%)	57 (74.0%)	34 (75.6%)	32 (68.1%)	0.922
Moderate to severe	46 (27.2%)	20 (26.0%)	11 (24.4%)	15 (31.9%)	
QoL VAS – median (IQR)^1^	6 (5–8)	6 (4–8)	8 (5–9)	6 (5–8)	0.225**
Pain VAS – median (IQR)^1^	2 (0–4)	2 (0–3)	3 (1–6)	2 (0–4)	0.001**
WPAI – Median (IQR)	5 (2–9)	4.5 (1.5–7.0)	4.5 (1.5–7.0)	7.5 (2.5–10.0)	0.126**

**ANOVA, *IQR* interquartile range, *BMI* body mass index, *FVC* forced vital capacity, *FEV*_*1*_ forced expiratory volume in 1 s, *DLCO* diffusion capacity of carbon monoxide, *TLC* total lung capacity, *PEF* peak expiratory flow, *pO*_*2*_ oxygen partial pressure, *pCO*_*2*_ carbon dioxide partial pressure, *pH* pondus hydrogenii (weight of hydrogen); *sO*_*2*_ saturation of oxygen, *CRP* C-reactive protein, *NT-proBNP* N-terminal pro-B-type natriuretic peptide, *AST* aspartate aminotransferase, *ALT* alanine aminotransferase, *FAS* fatigue assessment scale, *QoL* quality of life, *VAS* visual analog scale, *WPAI* work productivity and impairment score

### Patient-reported outcome measures (PROMs)

Quality of life (QoL), levels of pain, depression, anxiety, and work productivity were assessed using validated standard questionnaires HADS, PHQ-9, and GAD-7. Fatigue severity was measured using the Fatigue Assessment Scale (^©^ FAS Fatigue Assessment Scale: ild care foundation www.ildcare.nl, supplementary (suppl.) material for more details). A change of four points or 10% in the FAS score is considered the Minimal Important Difference (MCID) if performed at different time points [12–14]. Patients were also asked to rate their levels of fatigue before disease onset (pre-COVID-19) retrospectively within the last three months before COVID-19 and the difference between the two time points was calculated. Limitations in daily life were evaluated using a self-developed questionnaire based on the post-COVID-19 functional status scale (PCFS) [15] using a five-point Likert-scale covering the following topics: COVID-19-specific symptoms, pain or psychological burden, and daily physical performance. Results were categorized into improved, stable, and worsened (suppl. material).

The sociodemographic standards of the German statistical federal agency of statistics (destatis) were used to assess occupation and general and professional education [16].

### Immunophenotyping

Quantification of blood immune cell phenotypes of peripheral venous EDTA whole blood was performed using the BD Trucount™ System (Multitest™ 6-Color TBNK: CD3-FITC, CD16-PE, CD56-PE, CD45-PerCP-Cy5.5, CD4-Pe-Cy7, CD19-APC, CD8-APC-Cy7 (Trucount) with tubes; BD Biosciences cat #337,166) according to the manufacturer’s instructions. In brief, 20 μL of BD Multitest 6-Color TBNK antibody solution was mixed with 50 μL of whole blood in Trucount tubes. The solution was vortexed and incubated for 15 min at RT in the dark. Next, 450 μL of BD FACS Lysing Solution (1x), which was previously diluted 1:10 from the 10 × stock solution in distilled aqua, was added to the sample, vortexed, and incubated again for 15 min at RT in the dark to allow lysis of the erythrocytes. Samples were immediately measured on the LSR-II flow cytometer by recording 200,000 cell events and analyzed using FACSDiva software (version 8.0.1). The gating strategy is depicted in suppl Fig. 1.

### SARS-CoV-2 serology and neutralizing capacity

IgG, IgA, and IgM antibodies specific for the S1, S2, receptor-binding (RBD) domains and the nucleocapsid (N) antigen were detected in plasma samples using the SARS-CoV-2 Antigen Panel 1 IgG, IgM, and IgA assay (Millipore HC19SERM1-85 K-04, HC19SERA1-85 K-04, HC19SERG1-85 K-04) following the manufacturer’s instructions. EDTA plasma samples were diluted 1:200 in sample diluent and 25 µl was used for incubation with the bead mixture containing S1-, S2-, RBD-, and N-coated beads together with three positive and one negative bead control. After incubation and three washing steps, specific IgG, IgM, or IgA antibodies were detected by goat-anti-human Ig-specific PE-labeled secondary antibodies. The median fluorescence intensity (MFI) of > 50 beads was measured using a Bio-Rad200 system and Bio-Plex Manager 6.2 Software. Inhibition capacity of IgG antibodies against SARS-CoV-2 was measured as interference of patients’ antibodies between the spike protein of certain variants and the human angiotensin converting enzyme 2 (ACE2) using the Bio-Plex Pro Human SARS-CoV-2 Variant Neutralization Antibody 11-Plex Panel #12,016,897. The neutralizing capacity was quantified using a VSV-based pseudovirus luciferase neutralization assay with serial 1:25, 100, 400 1600 dilutions of plasma in triplicates [17]. Virus neutralization titers reducing infection by 50% (VNT50) were determined for each plasma sample.

### Statistical analysis

IBM SPSS Statistics 28.0 (IBM Corp, Armonk, NY, USA), STATA 13.0 (State Corp LP, College Station, Texas, USA), and PRISM V9.10 statistical software were used for analysis of the data. Categorical variables are stated as numbers (n) and percentages (%). Continuous variables are shown as median with interquartile ranges (IQR) unless indicated otherwise. For group comparisons, Fisher’s exact test, Chi-squared test, two-sided t-test, ANOVA, or Mann–Whitney U-test were used, as appropriate. Correlation of immunological and clinical symptoms was established by using Spearman’s correlations coefficient R. Univariate and multivariate linear regression with forward selection and pairwise deletion were calculated to assess correlates of fatigue with all clinical parameters. All reported p-values are tw -sided. *P*-values < 0·05 were considered statistically significant.

## Results

### Baseline characteristics

Table 1 summarizes baseline characteristics of all 176 convalescent COVID-19 patients stratified into prior non-hospitalized (non-hos), hospitalized non-ICU (non-ICU), and hospitalized ICU (ICU) patients. Refer to suppl. Table 1 for additional details.

Patients were assessed a median of 70 days (IQR 58–101) after initial symptom onset of COVID-19 without a significant difference between the groups. Symptom burden and clinical impairment characteristics during assessment are given in Table 2. Frequency of reported physical post-COVID-19 symptoms did not differ between the groups. In clinical phenotyping, PFTs showed lower median FVC, FEV_1_, DLCO, and TLC %/pre in hospitalized patients but remained within normal limits in all groups except for DLCO %/pre (67%/pre (IQR 51%–84%) in ICU patients (Table 2). ICU patients had lower absolute and adjusted 6MWD and lower paO_2_ and sO_2_ in capillary blood gas (CBG) analysis at rest. Tested blood parameters did not differ across groups except for slightly higher AST and NT-proBNP levels in ICU patients.

### Functional assessment

Approximately 25% of patients reported moderate to severe impairment on the depression and anxiety spectrum according to HADS, PHQ-9, and GAD-7 scores, which was unrelated to initial disease severity. Quality of life, pain level, work productivity, and results of the self-compiled COVID-19-questionnaire did also not differ between the groups.

**Fig. 1:**
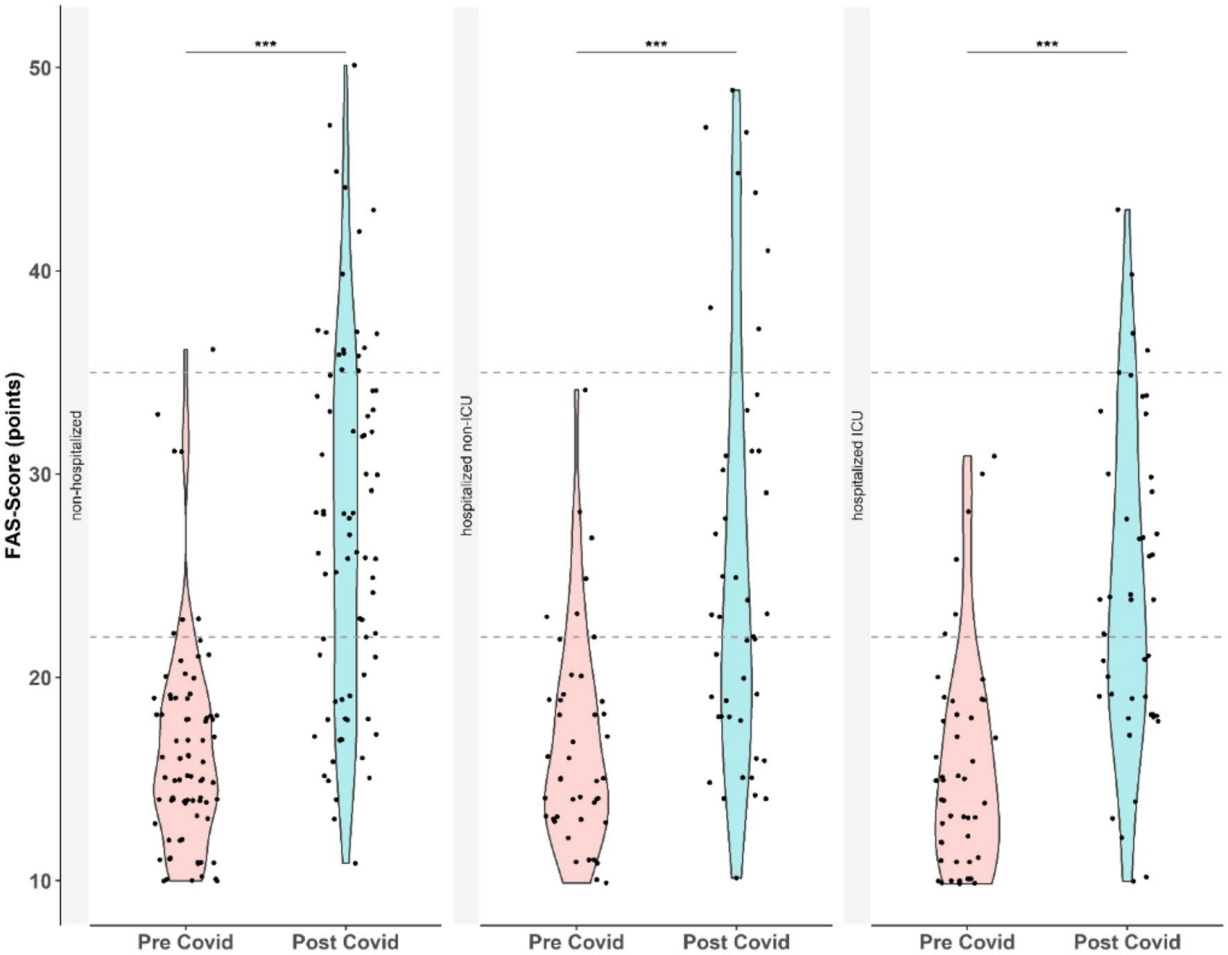
Violin plot of FAS score before and after COVID-19. Self-reported FAS score was obtained at follow-up visit. Pre-COVID was defined as within 3 months before COVID-19 and patients filled in the questionnaire retrospectively at their first visit to the clinic. All groups showed a statistically significant and clinically relevant increase of fatigue between the two time points. There was no significant difference between the three groups, neither before nor after COVID-19. *p < 0.05, **p < 0.01, ***p < 0.001, ****p < 0.0001.

Clinically relevant fatigue (FAS score of ≥ 22 points) was equally present in all three groups with an overall incidence of 29·5% (incidence ICU patients: 29·2%, non-ICU hospitalized patients: 20·0%, non-hospitalized patients: 35·9%). Extreme fatigue (FAS score of ≥ 35) was reported by 18·8% of the patients. Incidence for extreme fatigue was highest in non-hospitalized patients (23·1%) followed by hospitalized non-ICU patients (16%) and ICU patients (12·5%). All three groups reported clinically relevant increases in fatigue scores (Table 2). Severity of reported fatigue did not differ between COVID-19 patient groups (Fig. 1).

### Associations between immunological and clinical outcome parameters

#### Peripheral blood immune cell phenotypes

**Fig. 2:**
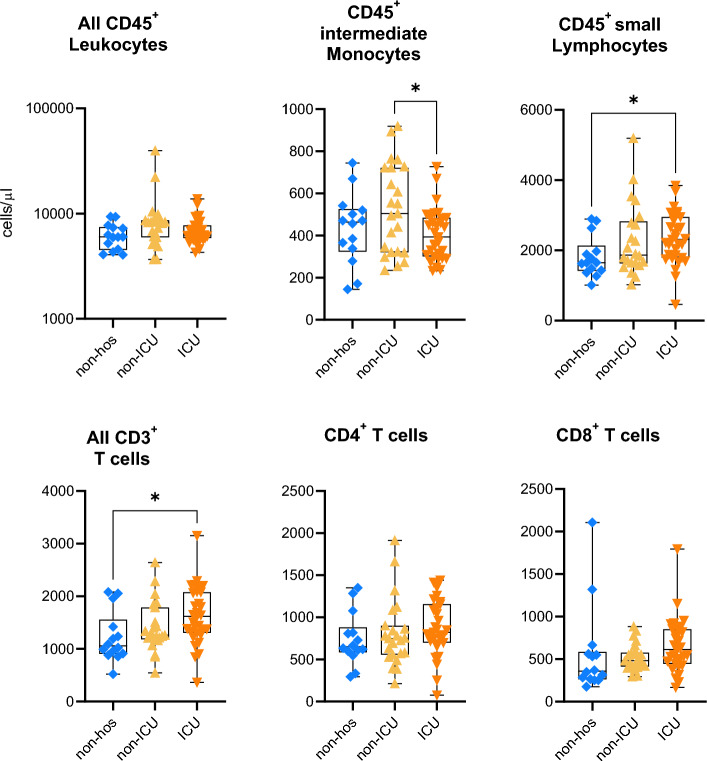
Immune cell numbers in blood of convalescent COVID-19 patients. Trucount analyses were used to analyze absolute numbers of immune cells in blood. *N* = 56 non-hospitalized (non-hos), *n* = 32 hospitalized non-intensive care unit (non-ICU), and *n* = 35 ICU convalescent COVID-19 patients. Statistical analysis: multigroup comparisons were performed using ANOVA test with Turkey multiple comparison test if possible, otherwise Kruskal–Wallis test and Dunn’s multiple comparison test were performed. *p < 0.05, **p < 0.01, ***p < 0.001, ****p < 0.0001.

In 123 convalescent patients (*n* = 56 non-hos, *n* = 32 non-ICU, *n* = 35 ICU), and *n* = 33 unexposed controls (UE)), immunophenotyping was performed by FACS analysis (Fig. 2, suppl. Figure 2). Post-acute absolute blood immune cell counts did not differ between unexposed controls and convalescent COVID-19 patients, independent of initial COVID-19 severity. Post-acute lymphocyte counts, in particular CD3^+^ T cells, were associated with initial COVID-19 severity (p < 0·05). Initially hospitalized patients had higher CD45^+^ intermediate monocyte counts compared with patients requiring ICU treatment (*p* < 0·05) (Fig. 2). CD19^+^ B cell, ^−^CD16/56^+^ NK cell, CD4^+^, and CD8^+^ T cell counts as well as activated CD56^+^CD4^+^ and CD8^+^ T cell counts did not differ between groups (Fig. 2, suppl. Figure 2).

To assess the correlation of immunological and clinical parameters, we selected a single parameter with the strongest effect size from each category (DLCO%/pre for PFT, pO2 for CBG analysis, 6MWD%/pre for exercise capacity and FAS score for psychosocial assessment) as dependent variables.

In non-hospitalized patients, fatigue severity (by FAS score) was positively correlated with lymphocyte and CD4^+^ T cell counts, and DLCO was positively correlated with total T cell counts (suppl. Figure 3).

In hospitalized, non-ICU patients, fatigue severity was positively correlated with leukocyte, granulocyte, and CD4^+^ T cell counts with FAS score. DLCO was negatively correlated with total T cell counts.

In ICU patients, fatigue severity directly correlated with total lymphocyte, and total CD3^+^ T cell and CD4^+^ T cell counts. Positive correlations were also detected for DLCO with CD4^−^CD8^−^ T cell counts, for adjusted 6MWD with CD3^−^CD16/56^+^ NK cell counts and for PaO_2_ with CD8^+^ T cell counts.

Of note, fatigue severity was positively correlated with CD4^+^ T cell counts across all patient groups (*r* = 0·703, *p* = 0·005 in non-hospitalized; *r* = 0·468, *p* = 0·050 in hospitalized non-ICU; *r* = 0·364, *p* = 0·048 in hospitalized ICU; suppl. Table 4). In addition, the Spearman’s correlation coefficient increased from ICU over non-ICU hospitalized to non-hospitalized patients in parallel to the increase of absolute CD4^+^ T cells, suggesting a potential link between CD4^+^ T cells and fatigue manifestation (Fig. 2, suppl. Figure 2 and suppl. Table 4). Details of the univariate linear regression analysis are provided in suppl. Table 3.

In the multivariate regression analysis, FAS score (as measure of fatigue severity) increased by 0·7 points for every 100 CD4^+^ T cells/µL whole blood (corrected r^2^ 0·599, *p* < 0·001) (suppl. Table 3).

#### Spike- and nucleocapsid-specific IgG antibodies and neutralizing capacities

Due to the early infection time point between spring and winter 2020 in this cohort, vaccination was not available and the D614G wildtype was the predominant variant. In terms of the humoral response to SARS-CoV-2 infection, in 124 patients, testing for SARS-CoV-2-specific IgG was performed including S1, S2, receptor-binding domain (RBD) and nucleocapsid (N) antigens (suppl. Table 5).

**Fig. 3:**
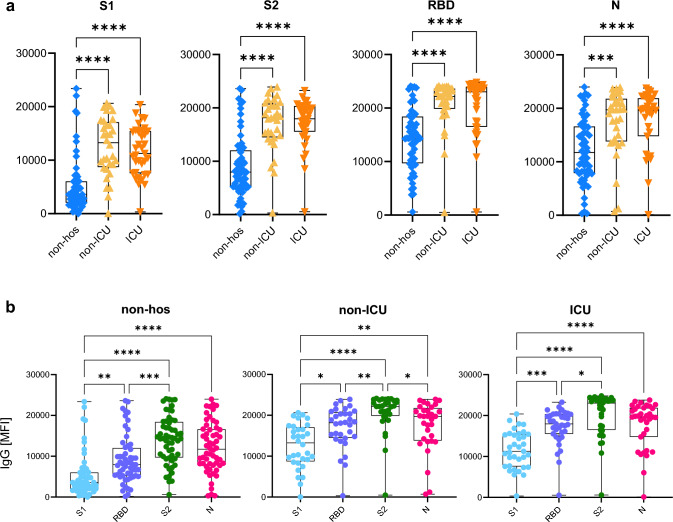
Level of IgG antibodies against SARS-CoV-2 S1-, S2-, RBD-, and N- antigens. (**a**, **b**) Luminex-based multiplex assays were used to measure IgG antibody levels against SARS-CoV-2, S1 domain, S2 domain, RBD, and N antigen in *n* = 56 non-hospitalized (non-hos), *n* = 32 hospitalized non- intensive care unit (non-ICU), and *n* = 35 ICU convalescent COVID-19 patients. Antibody levels are displayed as MFI. (**a**) Comparison of IgG levels between convalescent COVID-19 cohorts––non-hos, non-ICU, and ICU. (**b**) Comparison of S1-, RBD-, or S2-specific IgG levels separately in the three cohorts, non-hos, non-ICU, and ICU. Statistical analysis: multigroup comparisons were performed using Kruskal–Wallis test with Dunn’s multiple comparison test. *p < 0·05, **p < 0·01, ***p < 0·001, ****p < 0·0001.

**Fig. 4:**
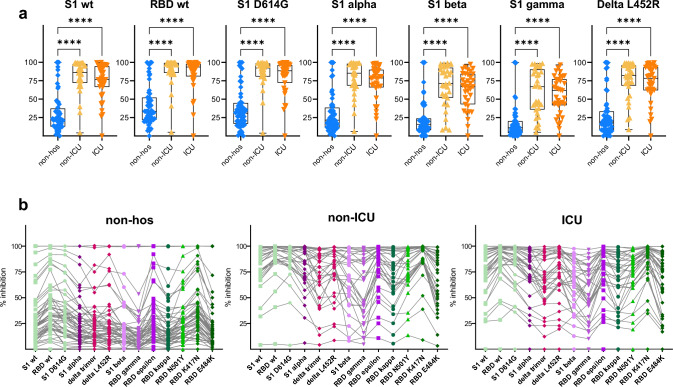
Inhibition capacity of IgG against several SARS-CoV-2 variants. (**a**, **b**) IgG inhibition capacity from *n* = 56 non-hospitalized (non-hos), *n* = 32 hospitalized non-intensive care unit (non-ICU), and *n* = 35 ICU convalescent COVID-19 patients was analyzed using Luminex-based multiplex assays. Neutralization is defined as interference of IgG antibodies in interaction between spike variants and the human ACE2. Neutralization is displayed as % inhibition. (**a**) Comparison between convalescent COVID-19 cohorts––non-hos, non-ICU, and ICU of a selection of wildtype, beta, gamma, and delta domains. (**b**) Intraindividual IgG inhibition capacity against all analyzed SARS-CoV-2 variants. Statistical analysis: multigroup comparisons were performed using Kruskal–Wallis test with Dunn’s multiple comparison test. Two-way repeated measures were performed using ANOVA test with Tukey multiple comparisons test. *p < 0·05, **p < 0·01, ***p < 0·001, ****p < 0·0001.

Therefore, a subgroup analysis of MHH patients was performed and IgG, IgA, and IgM levels specific for the S1, S2, RBD, and the N antigens were determined (Fig. 3a, suppl. Figure 4a). Except for three antibody-negative patients, relative IgG levels (as determined by MFI) were significantly higher in the hospitalized cohorts compared to the non-hospitalized group. All groups showed an increase of IgG levels from S1 lowest, RBD, N, and S2 highest (Fig. 4b). Levels of spike- and N-specific IgA and IgM antibodies were significantly lower in plasma of convalescent patients compared to IgG (suppl. Figure 4a). In a subgroup analysis of *n* = 51 samples, the neutralizing capacity in patient plasma aligned with S1-specific IgG levels, followed by S2- and RBD-specific antibodies indicating a collective contribution of all three spike regions in blocking infection by wildtype-spike pseudoviruses in vitro (suppl. Figure 4b). Spike-specific IgA and IgM antibodies correlated weakly with neutralization activity, suggesting that IgG assumes the primary role in neutralization. The potency of these spike-specific IgG antibodies in interfering with binding of spike domains of wildtype virus versus 10 various variants of concern was quantified (Fig. 4a, b). Across all spike domains, both wildtype and variants, the inhibition was significantly higher for plasma samples derived from hospitalized as opposed to non-hospitalized patients (*p* < 0,001). The direct comparison of all domains within the three groups in Fig. 4b unveiled the most efficient interference with the wildtype (wt) domains, S1 wt, RBD wt, and S1-D614G, and the lowest interference with the delta, gamma, and beta domains. Despite the high individual variability in IgG responses, plasma of individuals with > 85% interference with wildtype RBD domain (56/122, 45.9%) could also efficiently interfere with all other variants. This semi-quantitative effect was even more pronounced in the non-hospitalized group, in which only six of 56 patients (10.7%) reached this high level of interference and 95% of plasma samples contained antibodies insufficient for interfering with the beta domain, which represents the domain closest to the omicron variant. Overall, hospitalized non-ICU and ICU patients displayed higher inhibition capacity against all tested SARS-CoV-2 variants compared to non-hospitalized patients, suggesting that antibody levels as well as antibody inhibition capacity increase with COVID-19 severity (Fig. 4a).

No significant correlations were found between antibody levels of IgG and clinical outcome parameters including fatigue prevalence or severity (suppl. Figure 5).

## Discussion

Ten weeks following the onset of COVID-19, our initially immunonaïve cohort demonstrated a significant correlation between the fatigue severity and T cell phenotypes, regardless of the initial disease severity. Notably, even though non-hospitalized patients had fewer organ-specific complications, they reported comparable levels of impairment concerning fatigue, depression, anxiety, and overall quality of life.

In our cohort, hospitalized non-ICU patients showed higher monocyte counts than ICU patients 10 weeks after infection and, most strikingly, mean total lymphocyte and T cell counts were lowest in non-hospitalized patients. This is in line with reports of possible long-term monocyte, B, and T cell dysfunction in the peripheral blood of patients with PASC [11, 18]. Despite multiple reports of impaired or dysregulated immune responses during acute COVID-19 with differences between mild and severe disease [19], quality, longevity, and specificity of persisting immunity after recovery from COVID-19 are still poorly understood, especially with its potential impact on long-COVID.

All convalescent patients of our cohort displayed an antigen-dependent increase of S2- and N- compared to S1- and RBD-specific IgG antibodies. This can likely be explained by a stepwise antibody response, being firstly driven by cross-reactive memory B cells recognizing S2 and N epitopes and in a second step the generation of new antibody clones against the RBD and S1 domain [20]. Decreased levels of S1- and RBD-specific antibodies, which we have shown to be most potent neutralizers, suggest the need of a SARS-CoV-2 vaccination after infection. Moreover, this could promote antigen persistence, which has been identified as one potential driver of PASC [21]. Furthermore, a reduced in vitro antibody inhibition capacity of non-hospitalized patients especially against delta and beta variants of SARS-CoV-2 was found, which might explain breakthrough infection of patients with mild course of disease. The importance of repetitive vaccinations has been shown by Ruhl et al. [22]. In the multivariate analysis, CD4^+^ T cell counts were positively correlated with post-acute FAS score. Other studies have found altered T cell counts and function in patients with PACS but did not test the correlation with PROMs and fatigue in particular [18].

T cells are direct infection targets of SARS-CoV-2 and especially central/effector memory and cytotoxic CD4^+^ T cells prevail after infection with a half-life of up to ~ 200 days [23, 24]. Some reports suggest that COVID-19 can trigger sarcoidosis and sarcoidosis-like autoimmune conditions, which are associated with CD4^+^ T cell-mediated inflammation and feature a high prevalence of chronic fatigue [14, 25, 26]. In summary, our data indicate that fatigue in convalescent COVID-19 patients with PASC is linked with altered CD4^+^ T cell counts. If these fatigue-associated changes resolve over time remains an open questions.

The high psychological and mental burden of PACS is demonstrated by high prevalence of severe fatigue (FAS ≥ 35) and moderate to severe anxiety (24.4–31.0% of patients) and depression (20.0–25.6%) in our cohort. Overall quality of life and severity of depression symptoms were independently associated with fatigue in our cohort, which in part might be due to overlapping symptoms queried in the questionnaires.

A limitation of our study is the lack of information of clinical and functional parameters before the disease. Therefore, we cannot rule out prevalent organ dysfunction, especially lung function impairment. Also, we cannot exclude additional reasons for exertional dyspnea and impaired exercise tolerance, for example (respiratory) muscle weakness, which was only tested systematically in a subgroup of our cohort [27]. In addition, FAS for the time before COVID-19 was assessed retrospectively and might have been subject to recall bias.

One strength of our study, however, involves the early time point of the initial testing from May 2020 onwards allowing us to directly compare the immune phenotype of patients with PASC with healthy controls unexposed to SARS-CoV-2 or COVID-19 vaccines.

In conclusion, subjective disease burden as measured by PROMs was independent of disease severity. Our study identified an independent and direct correlation between severity of fatigue, as the most reported PASC symptom, and blood T cell counts paving the way for a clinically widely applicable blood biomarker of PACS-associated fatigue.

### Supplementary Information

Below is the link to the electronic supplementary material.

Supplementary file1 (PDF 325 KB)

Supplementary file2 (PDF 218 KB)

Supplementary file3 (PDF 110 KB)

Supplementary file4 (PDF 218 KB)

Supplementary file5 (PDF 86 KB)

Supplementary file6 (DOCX 67 KB)

## Data Availability

Files of Flow Cytometry Standard (FCS) are accessible at RepoMed (10.26068/mhhrpm/20231016-000).
