# Supplementary material for: Blood T cell phenotypes correlate with fatigue severity in post-acute sequelae of COVID-19
Source: Infection. 2023 Nov 4;52(2):513–24. doi: 10.1007/s15010-023-02114-8 (PMC10954951; doi:10.1007/s15010-023-02114-8)
Supplement: Supplementary file 1 — Supplementary file1 (PDF 325 KB) [file 15010_2023_2114_MOESM1_ESM.pdf]

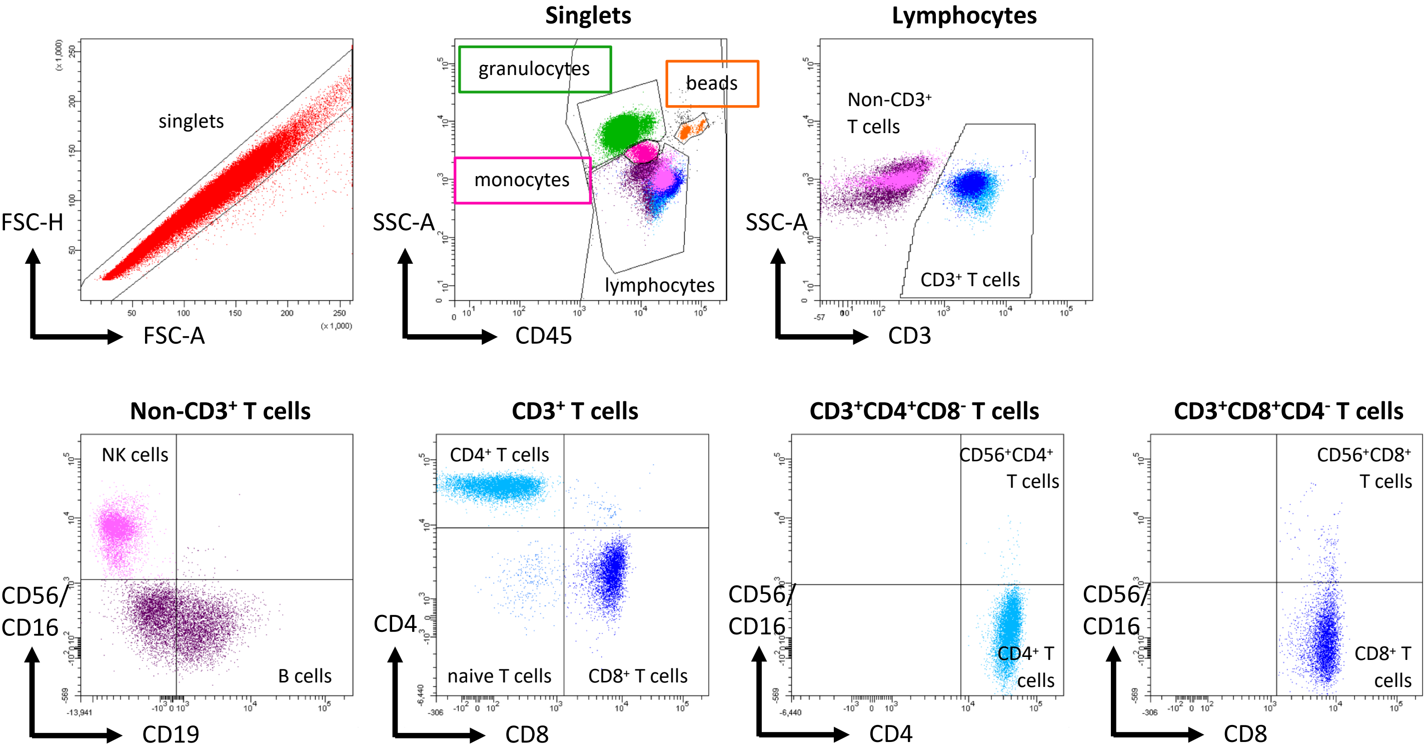

Supplementary Figure 1 Gating strategy for quantification of absolute cell counts of lymphocytes using the TruCount® kit (BD).

Events are gated in a FSC-A versus FSC-H plot to single cells. Beads, granulocytes, monocytes, and lymphocytes were identified from the single cells by plotting CD45 against SSC-A. Lymphocytes were then separated into CD3+ T cells and non-CD3+ T cells using a CD3-Gate. Non-CD3+ T cells are then gated to CD19+ B cells and CD56+/CD16+ NK cells, while the CD3+ T cells are divided into naive T cells (CD4-/CD8-), CD4+ T cells, and CD8+ T cells. CD3+CD4+ and CD3+CD8+ T cells, respectively, are separated into CD56+CD4+ and CD56+CD8+ T cells, respectively, based on CD56 expression. Absolute cell number (cells/µL) is determined by dividing the number of cellular events by the number of bead events and multiplying by TruCount bead concentration.
