# Supplementary material for: Blood T cell phenotypes correlate with fatigue severity in post-acute sequelae of COVID-19
Source: Infection. 2023 Nov 4;52(2):513–24. doi: 10.1007/s15010-023-02114-8 (PMC10954951; doi:10.1007/s15010-023-02114-8)
Supplement: Supplementary file 2 — Supplementary file2 (PDF 218 KB) [file 15010_2023_2114_MOESM2_ESM.pdf]

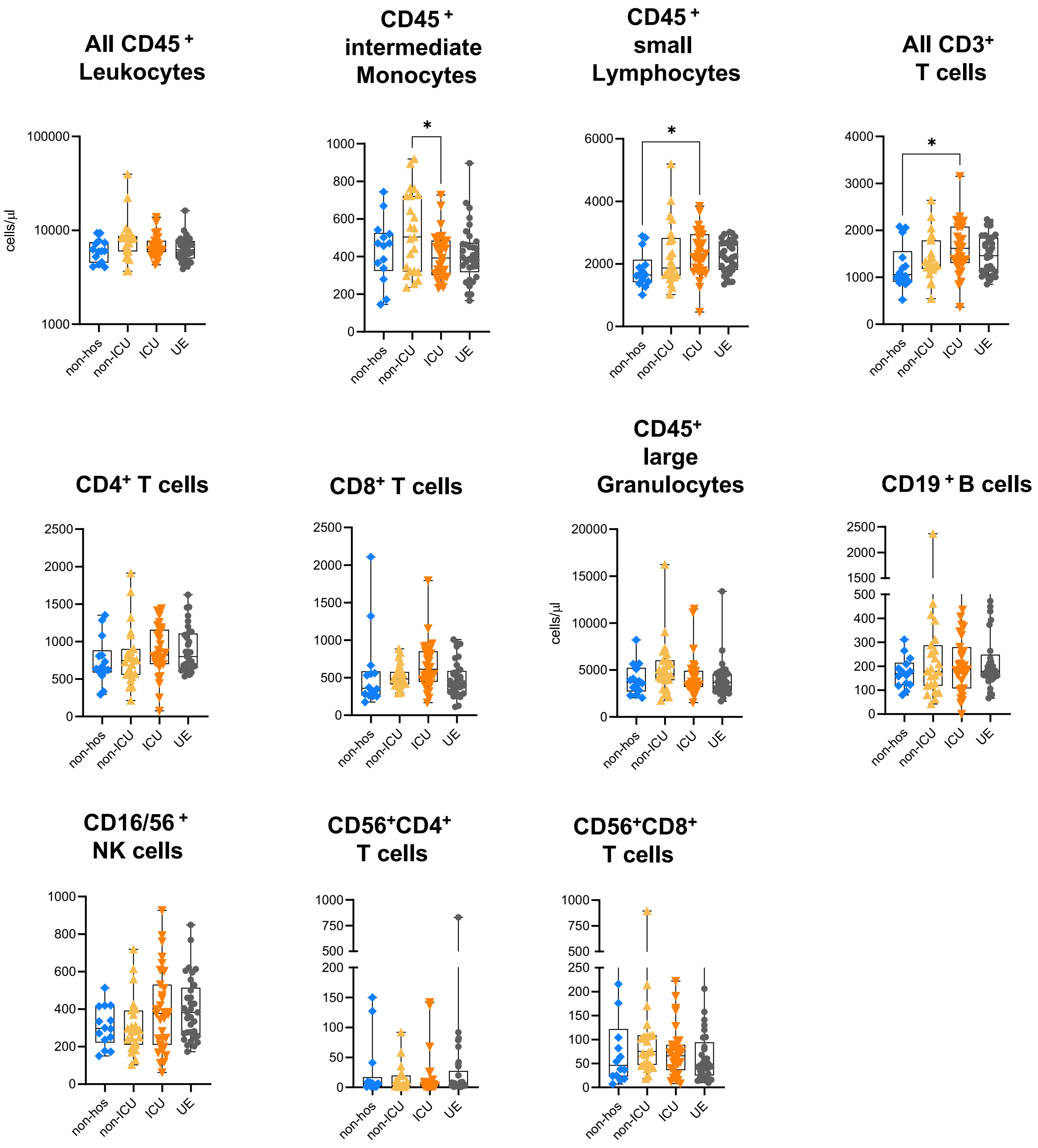


**Supplementary Figure 2 Immune cell numbers in blood of convalescent COVID-19 patients compared to unexposed donors**

TruCount analyses were used to analyze absolute numbers of immune cells in blood. n=56 non-hospitalized (non-hos), n=32 hospitalized non- intensive care unit (non-ICU) and n=35 ICU convalescent COVID-19 patients, UE: unexposed (n=33, 58% female, mean age 45·6 years (range 21·5 - 80·9 years), Statistical analysis: multigroup comparisons were performed using ANOVA test with Turkey multiple comparison test if possible, otherwise Kruskal–Wallis test with Dunn's multiple comparison test were performed. *p < 0·05, **p < 0·01, ***p < 0·001, ****p < 0·0001
