# Supplementary material for: Blood T cell phenotypes correlate with fatigue severity in post-acute sequelae of COVID-19
Source: Infection. 2023 Nov 4;52(2):513–24. doi: 10.1007/s15010-023-02114-8 (PMC10954951; doi:10.1007/s15010-023-02114-8)
Supplement: Supplementary file 3 — Supplementary file3 (PDF 110 KB) [file 15010_2023_2114_MOESM3_ESM.pdf]

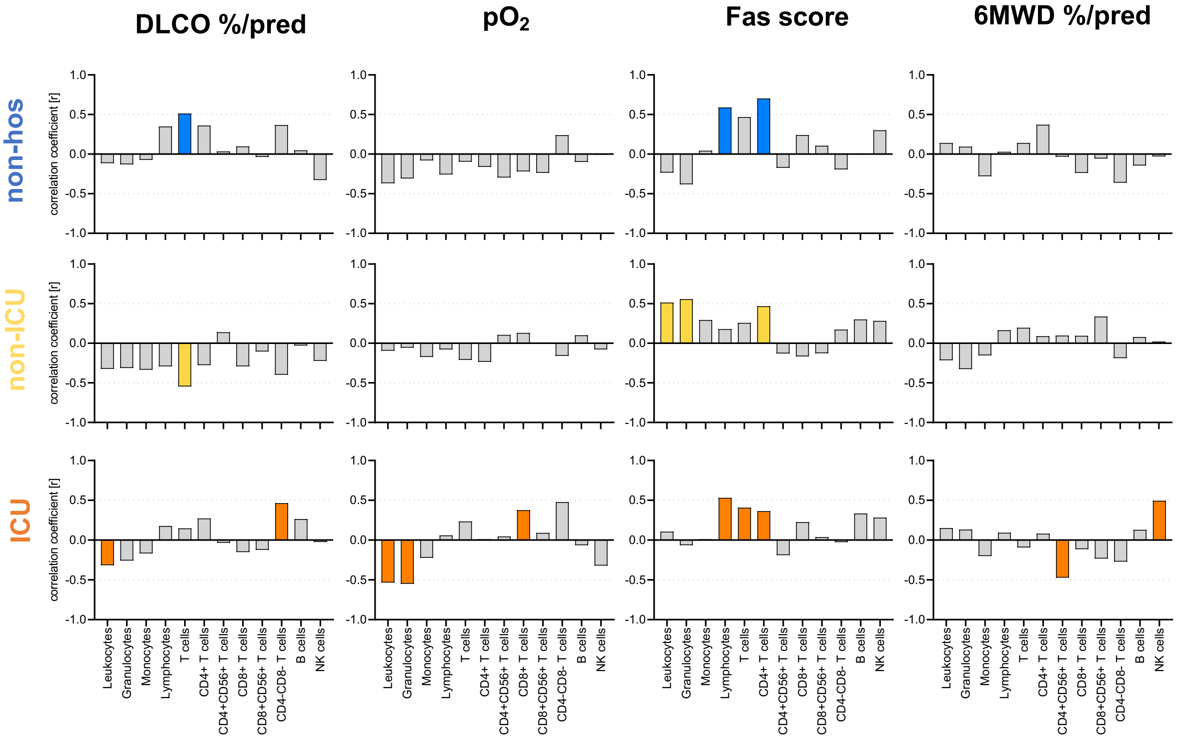

Supplementary Figure 3 Correlation of immune cell numbers with clinical parameters

Absolute numbers of immune cells in blood were analyzed using TruCount analyses and correlated to the clinical parameters percentage of the predicted Diffusion capacity of carbon monoxide (DLCO), oxygen partial pressure (pO2), fatigue (FAS score) and percentage of the predicted six-minute-walking-test distance (6MWD %/pred) . n=15 non-hospitalized (non-hos), n=22 hospitalized non- intensive care unit (non-ICU) and n=31 ICU convalescent COVID-19 patients were included. Colored bars represent significant results with $p \leq 0·05$. Dotted lines represent the Spearman-correlation coefficient (r) of 0·5 or -0·5. Statistical analysis: Spearman correlation.
