# Supplementary material for: Blood T cell phenotypes correlate with fatigue severity in post-acute sequelae of COVID-19
Source: Infection. 2023 Nov 4;52(2):513–24. doi: 10.1007/s15010-023-02114-8 (PMC10954951; doi:10.1007/s15010-023-02114-8)
Supplement: Supplementary file 5 — Supplementary file5 (PDF 86 KB) [file 15010_2023_2114_MOESM5_ESM.pdf]

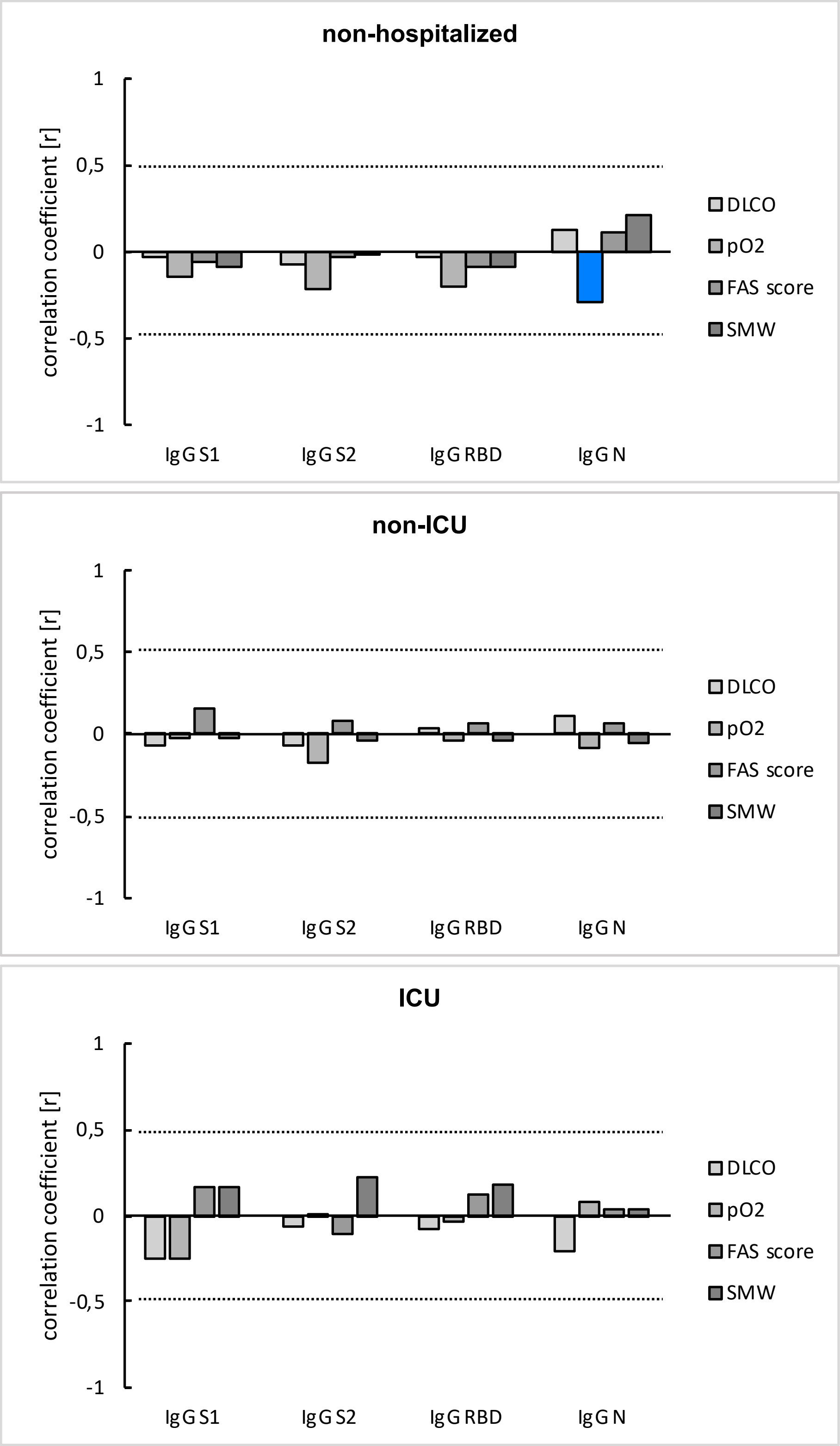

Supplemenatary Figure 5 Correlation of antibody level of IgG with clinical parameters

Absolute numbers of antibody IgG S1, IgG S2, IgG RBD and IgG N were analyzed using the SARS-CoV-2 Antigen Panel 1 IgG (Millipore, HC19SERG1-85K) and correlated to the clinical parameters percentage of predicted Diffusion capacity of carbon monoxide (DLCO), oxygen partial pressure (pO2), fatigue (Fas score) and percentage of predicted six-minute-walking-test (SMW). n=56 non-hos, n=31 non-ICU and n=35 ICU convalescent COVID-19 patients were included. Colored bars represent significant results with $p \leq 0{\cdot}05$ Dotted lines represent the Spearman-correlation coefficient (r) of 0·5 or -0·5. Statistical analysis: Spearman correlation.
