# Supplementary material for: Blood T cell phenotypes correlate with fatigue severity in post-acute sequelae of COVID-19
Source: Infection. 2023 Nov 4;52(2):513–24. doi: 10.1007/s15010-023-02114-8 (PMC10954951; doi:10.1007/s15010-023-02114-8)
Supplement: Supplementary file 6 — Supplementary file6 (DOCX 67 KB) [file 15010_2023_2114_MOESM6_ESM.docx]

**Supplementary tables and material**

**Blood T cell phenotypes correlate with fatigue severity in post-acute sequelae of COVID-19**

Isabell Pink^1,2, §^, Jan K. Hennigs^3,§^, Louisa Ruhl^4^, Andrea Sauer^1^, Lennart Boblitz^1^, Marie Huwe^3^, Jan Fuge^1,2^, Christine S. Falk^4^, Thomas Pietschmann^5^, Martina de Zwaan^6^, Antje Prasse^1^, Stefan Kluge^7^, Hans Klose^3^, Marius M. Hoeper^1,2^, Tobias Welte^1,2^

^1^Department of Respiratory Medicine, Hannover Medical School, Hannover, Germany

^2^Biomedical Research in Endstage and Obstructive Lung Disease Hannover (BREATH), German Center for Lung Research (DZL), Hannover, Germany

^3^Division of Respiratory Medicine, II. Department of Medicine, University Medical Center Hamburg-Eppendorf, 20251 Hamburg, Germany

^4^Institute of Transplant Immunology, Hannover Medical School, Hannover, Germany; German Center for Infection Research (DZIF), TTU-IICH, Germany

^5^Institute of Experimental Virology, TWINCORE Research Center, Hannover Medical School, Hannover, Germany

^6^Department of Psychosomatic Medicine and Psychotherapy, Hannover Medical School, Hannover, Germany

^7^Department of Intensive Care Medicine, University Medical Center Hamburg-Eppendorf, 20251 Hamburg, Germany

**Corresponding author**

Isabell Pink, MD

Hannover Medical School

Department of Respiratory Medicine

Carl-Neuberg-Str. 1

30625 Hannover

Germany

Phone: 0511 532 9314

E-Mail: [pink.isabell@mh-hannover.de](mailto:pink.isabell@mh-hannover.de)

^§^ shared first authorship

**Table of contents**

[**Supplementary Table 1 Baseline characteristics of non-hospitalized· hospitalized non-ICU and ICU patients** 2](#_Toc142380894)

[**Supplementary Table 2 Symptom burden and clinical impairment at follow-up** 4](#_Toc142380895)

[**Supplementary Table 3 Impact on Score of Fatigue Assessment Scale (FAS-Scale)** 7](#_Toc142380896)

[**Supplementary Table 4 Correlation of immunological and clinical parameters by Spearmen’s correlation** 8](#_Toc142380897)

[**Supplementary Table 5 Average level of antibodies at follow-up** 12](#_Toc142380898)

[**Characteristics of acute COVID-19 disease** 13](#_Toc142380899)

[**Limitations in dialy life - Questions compiled by the first authors** 14](#_Toc142380900)

[**Details of Patient-reported Outcome Measures** 15](#_Toc142380901)

# **Supplementary Table 1 Baseline characteristics of non-hospitalized· hospitalized non-ICU and ICU patients**

|  | **All**  **n=176** | **Non-hospitalized patients**  **n=78** | **Hospitalized non-ICU patients**  **n=50** | **Hospitalized ICU patients**  **n=48** | **P value (Hospitalized vs Non-hospitalized patients)** | **P value (Non-hospitalized vs Hospitalized non-ICU vs ICU patients)** |
| --- | --- | --- | --- | --- | --- | --- |
| **Site – n (%)** |  |  |  |  |  |  |
| - Hannover MHH | 124 (70·5%) | 56 (45·2%) | 32 (25·8%) | 36 (29·0%) | 0·728 | 0·462 |
| - Hamburg UKE | 52 (29·5%) | 22 (42·3%) | 18 (34·6%) | 12 (23·1%) |  |  |
| **Age – Median (IQR)** | 49·8 (38·7-58·9) | 45·0 (33·2-51·7) | 56·0 (48·2-65·4) | 54·1 (42·0-63·3) | **< 0·001** | **<0·001** |
| **Sex – n (%)** |  |  |  |  |  |  |
| - female | 79 (44·9%) | 47 (60·3%) | 18 (36·0%) | 14 (29·2%) | **< 0·001** |  |
| - male | 97 (55·1%) | 31 (39·7%) | 32 (64·0% | 34 (70·8%) |  |  |
| **Ethnic group** |  |  |  |  |  | 0·404 |
| - Caucasian | 172 (97·7) | 77 (98·7%) | 49 (98·0%) | 46 (95·8%) |  |  |
| - Asian | 2 (1·1%) | 1 (1·3%) | 0 (0·0%) | 1 (2·1%) |  |  |
| - African | 1 (0·5%) | 0 (0·0%) | 0 (0·0%) | 1 (2·1%) |  |  |
| - Hispanic | 1 (0·5%) | 0 (0·0%) | 1 (2·0% | 0 (0·0%) |  |  |
| **Family Status – n (%)** |  |  |  |  |  |  |
| - single | 49 (27·8%) | 30 (38·5%) | 9 (18·0%) | 10 (20·8%) | **0·044** | 0·228 |
| - married | 109 (61·9%) | 41 (52·6%) | 33 (66·0%) | 32 (66·7%) |  |  |
| - widowed | 5 (2·8%) | 1 (1·3%) | 2 (4%) | 2 (4·2%) |  |  |
| - divorced | 13 (7·4%) | 4 (5·1%) | 5 (10·0%) | 4 (8·3%) |  |  |
| **Education** |  |  |  |  |  |  |
| - low | 23 (13·1%) | 2 (2·6%) | 6 (12·0%) | 15 (31·3%) | **0·001** | **<0·001** |
| - intermediate | 77 (43·8%) | 35 (44·9%) | 25 (50·0%) | 17 (35·4%) |  |  |
| - high | 76 (43·2%) | 41 (52·6%) | 19 (38·0%) | 16 (33·3%) |  |  |
| **Smoking – n (%)** |  |  |  |  |  |  |
| - never | 99 (56·3%) | 42 (53·8%) | 28 (56·0%) | 29 (60·4%) | 0·271 | 0·585 |
| - active | 12 (6·8%) | 8 (10·3%) | 2 (4%) | 2 (4·2%) |  |  |
| - former | 65 (36·9%) | 28 (35·9%) | 20 (40%) | 17 (35·4%) |  |  |
| Packyears – Median (IQR) | 6 (4-15) | 5 (4-13) | 6 (3-18) | 15 (5-25) | 0·257 | 0·087 |
| **Exposure to Pollutants – n (%)** | 81 (46%) | 39 (50·0%) | 23 (46·0%) | 19 (39·6%) | 0·345 | 0·523 |
| **Vaccination – n (%)** |  |  |  |  |  |  |
| - Influenza | 72 (40·9%) | 25 (32·1%) | 26 (52·0%) | 21 (43·8%) | **0·033** | 0·073 |
| - Pneumococcus | 31 (17·6%) | 11 (14·1%) | 7 (14·0%) | 13 (27·1%) | 0·275 | 0·130 |
| **Comorbidities – n (%)** |  |  |  |  |  |  |
| - Diabetes | 21 (11·9%) | 1 (1·3%) | 6 (12·0%) | 14 (29·2%) | **< 0·001** | **< 0·001** |
| - COPD | 18 (10·2%) | 5 (6·4%) | 3 (6·0%) | 10 (20·8%) | 0·136 | **0·017** |
| - Cardiovascular diseases | 23 (13·1%) | 2 (2·6%) | 10 (20·0%) | 11 (22·9%) | **< 0·001** | **0·001** |
| - Hypertension | 44 (25·0%) | 7 (9·0%) | 16 (32·0%) | 21 (43·8%) | **< 0·001** | **< 0·001** |
| - Chronic renal failure | 5 (2·8%) | 0 (0%) | 2 (4·0%) | 3 (6·3%) | 0·043# | 0·103 |
| - Obesity (BMI ≥ 30kg/m^2^) | 42 (23·9%) | 10 (12·8%) | 13 (26·0%) | 19 (39·6%) | **0·002** | **0·003** |
| - Liver disease | 3 (1·7%) | 0 (0%) | 1 (2·0%) | 2 (4·2%) | 0·119# | 0·211 |
| - Depression | 9 (5·1%) | 1 (1·3%) | 3 (6·0%) | 5 (10·4%) | **0·040** | 0·073 |
| **Covid-19-related initial symptoms – n (%)** |  |  |  |  |  |  |
| - Loss of smell | 86 (48·9%) | 57 (73·1%) | 17 (34·0%) | 12 (25·0%) | **< 0·001** | **< 0·001** |
| - Dyspnea | 123 (69·9%) | 41 (52·6%) | 40 (80·0%) | 42 (87·5%) | **< 0·001** | **< 0·001** |
| - Dry Cough | 108 (61·4%) | 44 (56·4%) | 32 (64·0%) | 32 (66·7%) | 0·229 | 0·467 |
| - Fever | 132 (75·0%) | 45 (57·7%) | 44 (88·0%) | 43 (89·6%) | **< 0·001** | **< 0·001** |
| - Chest tightness | 77 (43·8%) | 37 (47·4%) | 25 (50·0%) | 15 (31·3%) | 0·379 | 0·118 |
| - Fatigue | 144 (81·8%) | 70 (89·7%) | 37 (74·0%) | 37 (77·1%) | **0·015** | **0·048** |
| - Headache | 92 (52·3%) | 49 (62·8%) | 20 (40·0%) | 23 (47·9%) | **0·012** | **0·032** |
| - Diarrhea | 38 (21·6%) | 16 (20·5%) | 10 (20·0%) | 12 (25%) | 0·756 | 0·795 |
| WHO Improvement Scale (Disease Onset) – Median (IQR) | 4 (2- 5) | 2 (2) | 4 (3-4) | 6 (5-7) | **< 0·001*** | **< 0·001**** |
| WHO Improvement Scale (Baseline Visit) – Median (IQR) | 2 (2-2) | 2 (2) | 2 (1-2) | 2 (2) | 0·899* | 0·066** |
| **Days from symptom onset to visit** |  | 63 (51·8-99·5) | 69·0 (60·3-87·3) | 81·0 (65·5-110·0) |  | 0·123** |
| **Days from Symptoms to Hospitalization – Median (IQR)** | - | - |  |  | - |  |
| **Mechanical Ventilation – n (%) – ICU only** | - | - |  | 30 (17% of all· 61·2% of ICU) | - |  |
| **ECMO – n (%) – ICU Only** | - | - | - | 8 (4·5% of all· 16·3% of ICU) | - |  |

*Mann-Whitney-U-Test; **ANOVA; Abbreviations: ICU: intensive care unit; IQR: interquartile range; COPD: chronic obstructive pulmonary disease; BMI: body mass index; WHO: world health organization; ECMO: Extra-corporal membrane oxygenation

# **Supplementary Table 2 Symptom burden and clinical impairment at follow-up**

| tin | **All**  **n=176** | **Non-hospitalized patients**  **n=78** | **Hospitalized non-ICU patients**  **n=50** | **Hospitalized ICU patients**  **n=48** | **P value (Hospitalized vs Non-hospitalized patients)** | **P value (Non-hospitalized vs Hospitalized non-ICU vs ICU patients)** |
| --- | --- | --- | --- | --- | --- | --- |
| **Days after symptom onset to first visit – median (IQR)** | 69·5 (58·0-101·0) | 63·0 (51·8-99·5) | 69 (60·3-87·3) | 81·0 (65·5-110·0) | 0·288 | 0·123** |
| **Vital parameters – median (IQR)** |  |  |  |  |  |  |
| - BMI in kg/cm^2^ | 26·7 (23·5-29·8) | 24·6 (21·6-28·1) | 27·8 (24·4-30·8) | 28·4 (24·9-32·2) | **< 0·001** | **< 0·001 **** |
| **Lung Function –median (IQR)** |  |  |  |  |  |  |
| - FVC %/predicted | 97·0 (81·5-107·8) | 102·0 (94·5-111·5) | 94·5 (85·3-108·3) | 80·0 (65·5-102·0) | **< 0·001** | **< 0·001**** |
| - FEV_1_ %/predicted | 95·0 (83·0-105·0) | 98·0 (89·0-106·5) | 99·5 (86·0-107·5) | 83·0 (71·0-99·5) | **< 0·001** | **< 0·001**** |
| - FVC/FEV_1_ %/predicted | 85·0 (78·0-96·0) | 82·0 (76·0-92·0) | 85·0 (78·0-97·3) | 88·0 (81·5-97·5) | 0·269 | **0·037**** |
| - DLCO %/predicted | 86·0 (71·0-98·5) | 91·0 (83·5-102·0) | 84·0 (68·3-102·0) | 67·0 (50·5-83·5) | **0·002** | **< 0·001**** |
| - TLC %/predicted | 96·0 (84·0-107·0) | 103·0 (94·5-110·0) | 94·5 (84·0-105·0) | 86·0 (73·0-98·5) | **0·001** | **< 0·001**** |
| - PEF %/predicted | 88·0 (75·3-103·8) | 91·0 (79·5-104·0) | 87·5 (78·5-103·0) | 85·0 (70·5-104·0) | 0·345 | 0·464 |
| **Capillary Blood Gases – Median (IQR)** |  |  |  |  |  |  |
| - pO_2_ in mm Hg | 85·0 (78·7-91·3) | 89·2 (83·5-93·2) | 89·1 (83·9-95·7) | 79·0 (76·2-85) | 0·061 | **0·011** |
| - pCO_2_ in mm Hg | 38·7 (36·0-41·0) | 37·1 (34·2-39·9) | 37·2 (35·2-40·3) | 39·5 (35·6-41·2) | 0·120 | 0·510 |
| - pH | 7·42 (7·40-7·44) | 7·43 (7·41-7·45) | 7·43 (7·41-7·45) | 7·42 (7·40-7·44) | 0·115 | 0·282 |
| - sO_2_ in % | 97·2 (96·4-98·0) | 97·7 (96·8-98·4) | 97·4 (96·5-98·1) | 96·4 (95·2-97·0) | 0·064 | **0·014** |
| **Six Minute Walking Test – Median (IQR)** |  |  |  |  |  |  |
| - Distance in m | 561 (482-615) | 585 (544-648) | 550 (452-610) | 483 (410-654) | **< 0·001** | **< 0·001**** |
| - Distance %/predicted | 91 (82-102) | 93 (84-103) | 92 (82-110) | 84 (74-94) | **0·006** | **0·011**** |
| - s_p_O_2_ (delta) | -1 (-3–0) | 0·0 (-2·0–1·0) | -1·0 (-3·0-0·8) | -2·0 (-5·0-0·0) | 0·990 | 0·053 |
| - Pulse pre/post in bpm | 83/113 (73/98-96/126) | 83/114 (74/101- 94/129) | 84/110 (70/98-97/126) | 83/112 (69/97-98/123) | 0·070/0·757 | 0·958/0·114 |
| - Pulse (delta) | 31 (19–39) | 31·0 (22·0-45·0) | 30·5 (19·3-37·8) | 27·0 (13·0-36·0) | 0·879 | 0·069 |
| - BORG-Scale pre/post | 0/3 (0/1-2/4) | 0/3 (0/1-2/4) | 0/2 (0/0-2/3) | 0/3 (0/1-2/4) | 0·087/0·313 | 0·730/0·125 |
| - BORG-Scale (delta) | 2 (0-3) | 2·0 (0·5-3·0) | 1·0 (0·0-2·0) | 2·0(0·0-3·0 | 0·148 | 0·081 |
| **Blood markers** |  |  |  |  |  |  |
| - CRP in mg/l (n=141) | 2·0 (1·1-2·7) | 2·0 (0·8-2·0) | 2·0 (1·7-3·9) | 2·0 (0·9-3·0) | **0·028** | 0·110** |
| - NTproBNP in ng/l (n=57) | 74 (34-140) | 49·5 (34·0-95·8) | 57·0 (34·0-108·8) | 205·0 (87·0-1064·0) | **0·001** | **0·005**** |
| - Creatinine in µmol/l (n=142) | 76 (66-86) | 75·0 (66·8-82·7) | 77·9 (67·6-87·3) | 74·0 (63·0-84·1) | 0·097 | 0·332** |
| - AST in U/l (n=141) | 23 (18-29) | 21·0 (17·0-26·0) | 26·0 (19·3-31·5) | 22·0 (18·0-32·0 | **0·040** | 0·113** |
| - ALT in U/l (n=142) | 24 (18-37) | 20·0 (16·8-29·3) | 26·0 (20·0-39·5) | 26·0 (18·0-44·0) | 0·053 | 0·157** |
| **Post-COVID symptoms – n (%)** |  |  |  |  |  |  |
| - Chest tightness | 46 (26·1%) | 26 (33·3%) | 12 (24·0%) | 8 (16·7%) | 0·053 | 0·109 |
| - Cough | 46 (26·1%) | 25 (32·1%) | 7 (14·0%) | 14 (29·2%) | 0·111 | 0·065 |
| - Fever | 7 (4·0%) | 2 (·62%) | 3 (6·0%) | 2 (4·2%) | 0·392 | 0·622 |
| - Headache | 52 (29·5%) | 28 (35·9%) | 11 (22·0%) | 13 (27·1%) | 0·099 | 0·221 |
| **Quality of life and subjective condition** |  |  |  |  |  |  |
| - FAS – Median (IQR) |  |  |  |  |  |  |
| - - before disease onset | 15 (13-19) | 15·0 (13·8-19·0) | 15·0 (13·0-19·0) | 15·0 (11·0-19·0) | 0·445 | 0·599** |
| - - visit #1 | 25 (18-33) | 28·0 (19·0-34·5) | 23·0 (18·0-31·0) | 24·0 (18·0-30·0) | 0·946 | 0·151** |
| - - difference (delta) | 8 (2-17) | 12·0 (2·5-18·0) | 6·5 (2·0-15·0) | 6·5 (2·8-15·0) | 0·099 | 0·236** |
| - FAS Categories at first visit – n (%) |  |  |  |  |  |  |
| - - no fatigue (FAS <22) | 61 (36·5%) | 23 (29·9%) | 18 (40·9%) | 20 (43·5%) |  | 0·412 (Chi) |
| - - fatigue (FAS ≥22-34) | 73 (43·7%) | 35 (45·5%) | 18 (40·9%) | 20 (43·5%) | 0·167 |  |
| - - extreme fatigue (FAS ≥35) | 33 (19·8%) | 19 (24·7%) | 8 (18·2%) | 6 (13·0%) |  |  |
| - Depression – n (%) |  |  |  |  |  |  |
| - - None to minor | 132 (78·1%) | 61 (79·2%) | 36 (80·0%) | 35 (74·5% | 0·733 | 0·638 |
| - - Moderate to severe | 37 (21·9%) | 16 (20·8%) | 9 (20·0%) | 12 (25·6%) |  |  |
| - Anxiety – n (%) |  |  |  |  |  |  |
| - - None to minor | 123 (72·8%) | 57 (74·0%) | 34 (75·6%) | 32 (68·1%) | 0·946 | 0·922 |
| - - Moderate to severe | 46 (27·2%) | 20 (26·0%) | 11 (24·4%) | 15 (31·9%) |  |  |
| - QoL-VAS – median (IQR)^1^ | 6 (5-8) | 6 (4-8) | 8 (5-9) | 6 (5-8) | 0·138 | 0·225** |
| - Pain-VAS – median (IQR)^1^ | 2 (0-4) | 2 (0-3) | 3 (1-6) | 2 (0-4) | 0·195* | **0·001**** |
| **Occupation and Productivity** |  |  |  |  |  |  |
| - employment – n (%) |  |  |  |  |  | **<0·001** |
| - - full time employed | 103 (58·5%) | 52 (66·7%) | 27 (54·0%) | 24 (40·0%) | - |  |
| - - part time employed | 22 (12·5%) | 14 (17·9%) | 4 (8·0%) | 4 (6·7%) |  |  |
| - - unemployed | 37 (21·0%) | 5 (6·4%) | 15 (30·0%) | 17 (28·3%) |  |  |
| - - other | 14 (8%) | 7 (9·0%) | 4 (8%) | 3 (6·3%) |  |  |
| - WPAI – Median (IQR) | 5 (2-9) | 4·5 (1·5-7·0) | 4·5 (1·5-7·0) | 7·5 (2·5-10·0) | 0·567 | 0·126** |
| **Self-compiled Covid-19-Questions – n (%)** |  |  |  |  |  |  |
| - Covid-19-caused daily life restrictions (1) |  |  |  |  |  |  |
| - - improved | 82 (47·4%) | 32 (41·0%) | 29 (60·4%) | 21 (44·7%) | 0·162 | 0·179 (Chi) |
| - - stable | 50 (28·9%) | 28 (35·9%) | 10 (20·8%9 | 12 (25·5%) |  |  |
| - - worsened | 41 (23·7%) | 18 (23·1%) | 9 (18·8%) | 14 (29·8%) |  |  |
| - change in fitness (2) |  |  |  |  |  |  |
| - - improved | 60 (36·1%) | 26 (34·2%) | 17 (38·6%) | 17 (37·0%) | 0·233 | 0·106 (Chi) |
| - - stable | 70 (42·2%) | 37 (48·7%) | 12 (27·3%) | 21 (45·7%) |  |  |
| - - worsened | 36 (21·7%) | 13 (17·1%) | 15 (34·1%) | 8 (17·4%) |  |  |
| - change in psychological burden (3) |  |  |  |  |  |  |
| - - improved | 27 (16·3%) | 10 (13·2%) | 9 (20·5%) | 8 (17·4%) | 0·104 | 0·273 (Chi) |
| - - stable | 56 (33·7) | 32 (42·1%) | 10 (22·7%) | 14 (30·4%) |  |  |
| - - worsened | 83 (50·0%) | 34 (44·7%) | 25 (56·8%) | 24 (52·2%) |  |  |
| - ongoing burden of symptoms (4) |  |  |  |  |  |  |
| - - improved | 45 (27·4%) | 22 (28·9%) | 10 (23·8%) | 13 (28·3%) | 0·421 | 0·717 (Chi) |
| - - stable | 72 (43·9%) | 36 (47·4%) | 17 (40·5%) | 19 (41·3%) |  |  |
| - - worsened | 47 (28·7%) | 18 (23·7%) | 15 (35·7%) | 14 (30·4) |  |  |

*Mann-Whitney-U-Test; ** ANOVA; Abbreviations: IQR: interquartile range; BMI: body mass index; FVC: forced vital capacity; FEV_1_: forced expiratory volume in 1 second; DLCO: Diffusion capacity of carbon monoxide; TLC: total lung capacity; PEF: peak expiratory flow; pO_2_: oxygen partial pressure; pCO_2_: carbon dioxide partial pressure; pH: pondus Hydrogenii (weight of hydrogen); sO_2_: saturation of oxygen; CRP: C-reactive protein; NTproBNP: N-terminal pro-B-type natriuretic peptide; AST: Aspartate aminotransferase; ALT: Alanine aminotransferase; FAS: Fatigue Assessment Scale; QoL: Quality of life; VAS: visual analog scale; WPAI: Work Productivity and Impairment Score

# **Supplementary Table 3 Impact on Score of Fatigue Assessment Scale (FAS-Scale)**

|  | **Univariate Regression** | | | **Multivariate Regression** | | |
| --- | --- | --- | --- | --- | --- | --- |
| **Parameter** | **Regression coefficient B** | **95% Confidence interval** | ***P value*** | **Regression coefficient B** | **95% Confidence Interval** | **p-value** |
| **Sex** | 5·697 | 3·009 - 8·384 | **<0·001** |  |  |  |
| **Age** | -0·029 | -0·124 - 0·067 | 0·556 |  |  |  |
| **pO2** | 0·021 | -0·134 – 0·176 | 0·788 |  |  |  |
| **DLCO %/predicted** | 0·045 | -0·020 – 0·109 | 0·175 |  |  |  |
| **Depression** | 9·237 | 3·192 - 15·282 | **0·003** |  |  |  |
| **WHO Scale** | -0·714 | -1·660 - 0·232 | 0·138 |  |  |  |
| **BMI** | 0·199 | -0·095 - 0·494 | 0·184 |  |  |  |
| **6MWD % predicted** | -0·084 | -0·165 - -0·004 | **0·041** |  |  |  |
| **6MWT Pulse delta** | -0·080 | -0·151 - -0·009 | **0·027** |  |  |  |
| **6MWT SO_2_ delta** | 0·027 | -0·158 - 0·213 | 0·770 |  |  |  |
| **6MWT BORG-Scale delta** | 1·655 | 0·812 - 2·499 | **<0·001** |  |  |  |
| **Post-COVID Fever** | 3·129 | -4·389 - 10·648 | 0·412 |  |  |  |
| **Post-COVID Chest tightness** | 8·008 | 5·098 - 10·919 | **<0·001** |  |  |  |
| **Post-COVID Headache** | 6·181 | 3·271 - 9·091 | **<0·001** |  |  |  |
| **Post-COVID-cough** | 4·236 | 1·144 - 7·329 | **0·008** |  |  |  |
| **QoL-VAS median** | -2·742 | -3·340 - -2·144 | **<0·001** | -1·692 | -2·817 - -0·567 | **0·004** |
| **HADS-A-Score** | 1·443 | 1·096 - 1·791 | **<0·001** |  |  |  |
| **HADS-D-Score** | 1·414 | 1·109 - 1·719 | **<0·001** | 0·734 | 0·145 – 1·323 | **0·016** |
| **WPAI-Score** | 1·153 | 0·741 - 1·564 | **<0·001** |  |  |  |
| **Pain VAS** | 1·416 | 0·720 - 2·113 | **<0·001** |  |  |  |
| **Lymphocytes** | 0·000 | 0·000 - 0·001 | 0·320 |  |  |  |
| **CD3+ T cells** | 0·005 | 0·000 - 0·010 | **0·032** |  |  |  |
| **CD4+ T cells** | 0·009 | 0·002 - 0·016 | **0·008** | 0·007 | 0·002 – 0·013 | **0·013** |

Abbreviations: pO_2_: oxygen partial pressure; DLCO: Diffusion capacity of carbon monoxide; WHO: World Health Organisation; 6MWT: Six-minute walking test; 6MWD: 6MWT distance; SO_2_: saturation of oxygen; COVID: Coronavirus disease 2019; QoL: Quality of life; VAS: Visual analogue scale; HADS-A/D: Hospital Anxiety and Depression Scale Anxiety / Depression; WPAI: Work Productivity and Impairment Score

# **Supplementary Table 4 Correlation of immunological and clinical parameters by Spearmen’s correlation**

|  | | | | DLCO % of predicted | pO_2_ | FAS Score | Six-minute-walk distance % of predicted |
| --- | --- | --- | --- | --- | --- | --- | --- |
| **Non-Hospitalized patients**                                                                                                                                                            **Hospitalised Non-ICU patients**                                                                                                                                                            **Hospitalised ICU patients** | | **tc_lymphcytes** | Correlationscoeffizient | 0·349 | -0·257 | **0·589*** | 0·029 |
|  |  |  | Significance (two-sided) | 0·203 | 0·355 | 0·027 | 0·919 |
|  |  |  | N | 15 | 15 | 14 | 15 |
|  |  | **tc_cd3** | Correlationscoeffizient | 0·512 | -0·096 | 0·467 | 0·141 |
|  |  |  | Significance (two-sided) | 0·051 | 0·732 | 0·092 | 0·615 |
|  |  |  | N | 15 | 15 | 14 | 15 |
|  |  | **tc_cd4** | Correlationscoeffizient | 0·361 | -0·161 | **0·703**** | 0·370 |
|  |  |  | Significance (two-sided) | 0·186 | 0·567 | 0·005 | 0·174 |
|  |  |  | N | 15 | 15 | 14 | 15 |
|  |  | **tc_cd4_cd8** | Correlationscoeffizient | 0·367 | 0·239 | -0·192 | -0·361 |
|  |  |  | Significance (two-sided) | 0·179 | 0·390 | 0·511 | 0·186 |
|  |  |  | N | 15 | 15 | 14 | 15 |
|  |  | **tc_cd8** | Correlationscoeffizient | 0·098 | -0·218 | 0·240 | -0·238 |
|  |  |  | Significance (two-sided) | 0·729 | 0·435 | 0·410 | 0·393 |
|  |  |  | N | 15 | 15 | 14 | 15 |
|  |  | **tc_cd56_cd16** | Correlationscoeffizient | -0·327 | -0·007 | 0·300 | -0·030 |
|  |  |  | Significance (two-sided) | 0·234 | 0·980 | 0·298 | 0·914 |
|  |  |  | N | 15 | 15 | 14 | 15 |
|  |  | **tc_cd56_cd16_cd19** | Correlationscoeffizient | -0·065 | 0·030 | 0·107 | **-0·657**** |
|  |  |  | Significance (two-sided) | 0·819 | 0·914 | 0·715 | 0·008 |
|  |  |  | N | 15 | 15 | 14 | 15 |
|  |  | **tc_cd19** | Correlationscoeffizient | 0·047 | -0·100 | -0·002 | -0·145 |
|  |  |  | Significance (two-sided) | 0·869 | 0·723 | 0·994 | 0·606 |
|  |  |  | N | 15 | 15 | 14 | 15 |
|  |  | **tc_granulocytes** | Correlationscoeffizient | -0·129 | -0·307 | -0·381 | 0·095 |
|  |  |  | Significance (two-sided) | 0·647 | 0·265 | 0·178 | 0·737 |
|  |  |  | N | 15 | 15 | 14 | 15 |
|  |  | **tc_monocytes** | Correlationscoeffizient | -0·073 | -0·079 | 0·043 | -0·279 |
|  |  |  | Significance (two-sided) | 0·795 | 0·781 | 0·884 | 0·313 |
|  |  |  | N | 15 | 15 | 14 | 15 |
|  |  | **tc_cd56** | Correlationscoeffizient | 0·035 | -0·296 | -0·172 | -0·033 |
|  |  |  | Significance (two-sided) | 0·901 | 0·284 | 0·557 | 0·906 |
|  |  |  | N | 15 | 15 | 14 | 15 |
|  |  | **tc_cd8_cd58** | Correlationscoeffizient | -0·036 | -0·236 | 0·106 | -0·055 |
|  |  |  | Significance (two-sided) | 0·899 | 0·398 | 0·719 | 0·844 |
|  |  |  | N | 15 | 15 | 14 | 15 |
|  |  | **tc_all_cd45** | Correlationscoeffizient | -0·114 | -0·368 | -0·234 | 0·141 |
|  |  |  | Significance (two-sided) | 0·685 | 0·177 | 0·421 | 0·615 |
|  |  |  | N | 15 | 15 | 14 | 15 |
|  |  | **tc_lymphcytes** | Correlationscoeffizient | -0·290 | -0·077 | 0·179 | 0·164 |
|  |  |  | Significance (two-sided) | 0·202 | 0·734 | 0·478 | 0·476 |
|  |  |  | N | 21 | 22 | 18 | 21 |
|  |  | **tc_cd3** | Correlationscoeffizient | **-0·545*** | -0·209 | 0·257 | 0·195 |
|  |  |  | Significance (two-sided) | 0·011 | 0·351 | 0·303 | 0·397 |
|  |  |  | N | 21 | 22 | 18 | 21 |
|  |  | **tc_cd4** | Correlationscoeffizient | -0·275 | -0·234 | 0·468 | 0·089 |
|  |  |  | Significance (two-sided) | 0·228 | 0·295 | 0·050 | 0·701 |
|  |  |  | N | 21 | 22 | 18 | 21 |
|  |  | **tc_cd4_cd8** | Correlationscoeffizient | -0·398 | -0·159 | 0·173 | -0·187 |
|  |  |  | Significance (two-sided) | 0·074 | 0·479 | 0·494 | 0·416 |
|  |  |  | N | 21 | 22 | 18 | 21 |
|  |  | **tc_cd8** | Correlationscoeffizient | -0·291 | 0·131 | -0·165 | 0·094 |
|  |  |  | Significance (two-sided) | 0·201 | 0·561 | 0·513 | 0·686 |
|  |  |  | N | 21 | 22 | 18 | 21 |
|  |  | **tc_cd56_cd16** | Correlationscoeffizient | -0·222 | -0·078 | 0·282 | 0·023 |
|  |  |  | Significance (two-sided) | 0·333 | 0·730 | 0·257 | 0·922 |
|  |  |  | N | 21 | 22 | 18 | 21 |
|  |  | **tc_cd56_cd16_cd19** | Correlationscoeffizient | 0·014 | 0·188 | -0·258 | 0·104 |
|  |  |  | Significance (two-sided) | 0·951 | 0·401 | 0·301 | 0·652 |
|  |  |  | N | 21 | 22 | 18 | 21 |
|  |  | **tc_cd19** | Correlationscoeffizient | -0·030 | 0·101 | 0·301 | 0·079 |
|  |  |  | Significance (two-sided) | 0·898 | 0·654 | 0·225 | 0·733 |
|  |  |  | N | 21 | 22 | 18 | 21 |
|  |  | **tc_granulocytes** | Correlationscoeffizient | -0·313 | -0·055 | **0·558*** | -0·326 |
|  |  |  | Significance (two-sided) | 0·168 | 0·809 | 0·016 | 0·149 |
|  |  |  | N | 21 | 22 | 18 | 21 |
|  |  | **tc_monocytes** | Correlationscoeffizient | -0·335 | -0·172 | 0·295 | -0·153 |
|  |  |  | Significance (two-sided) | 0·138 | 0·443 | 0·235 | 0·509 |
|  |  |  | N | 21 | 22 | 18 | 21 |
|  |  | **tc_cd56** | Correlationscoeffizient | 0·139 | 0·106 | -0·129 | 0·098 |
|  |  |  | Significance (two-sided) | 0·549 | 0·639 | 0·609 | 0·671 |
|  |  |  | N | 21 | 22 | 18 | 21 |
|  |  | **tc_cd8_cd58** | Correlationscoeffizient | -0·105 | 0·007 | -0·126 | 0·338 |
|  |  |  | Significance (two-sided) | 0·652 | 0·974 | 0·618 | 0·134 |
|  |  |  | N | 21 | 22 | 18 | 21 |
|  |  | **tc_all_cd45** | Correlationscoeffizient | -0·322 | -0·094 | **0·514*** | -0·214 |
|  |  |  | Significance (two-sided) | 0·154 | 0·678 | 0·029 | 0·352 |
|  |  |  | N | 21 | 22 | 18 | 21 |
|  |  | **tc_lymphcytes** | Correlationscoeffizient | 0·178 | 0·059 | **0·530**** | 0·093 |
|  |  |  | Significance (two-sided) | 0·355 | 0·754 | 0·003 | 0·644 |
|  |  |  | N | 29 | 31 | 30 | 27 |
|  |  | **tc_cd3** | Correlationscoeffizient | 0·148 | 0·234 | **0·408*** | -0·093 |
|  |  |  | Significance (two-sided) | 0·445 | 0·205 | 0·025 | 0·645 |
|  |  |  | N | 29 | 31 | 30 | 27 |
|  |  | **tc_cd4** | Correlationscoeffizient | 0·273 | 0·011 | **0·364*** | 0·081 |
|  |  |  | Significance (two-sided) | 0·152 | 0·954 | 0·048 | 0·687 |
|  |  |  | N | 29 | 31 | 30 | 27 |
|  |  | **tc_cd4_cd8** | Correlationscoeffizient | 0·464 | **0·477**** | -0·026 | -0·270 |
|  |  |  | Significance (two-sided) | 0·011 | 0·007 | 0·891 | 0·173 |
|  |  |  | N | 29 | 31 | 30 | 27 |
|  |  | **tc_cd8** | Correlationscoeffizient | -0·150 | **0·376*** | 0·226 | -0·115 |
|  |  |  | Significance (two-sided) | 0·439 | 0·037 | 0·230 | 0·567 |
|  |  |  | N | 29 | 31 | 30 | 27 |
|  |  | **tc_cd56_cd16** | Correlationscoeffizient | -0·024 | -0·321 | 0·282 | **0·494**** |
|  |  |  | Significance (two-sided) | 0·901 | 0·078 | 0·131 | 0·009 |
|  |  |  | N | 29 | 31 | 30 | 27 |
|  |  | **tc_cd56_cd16_cd19** | Correlationscoeffizient | 0·446 | 0·065 | -0·044 | 0·129 |
|  |  |  | Significance (two-sided) | 0·015 | 0·730 | 0·816 | 0·521 |
|  |  |  | N | 29 | 31 | 30 | 27 |
|  |  | **tc_cd19** | Correlationscoeffizient | 0·264 | -0·064 | 0·334 | 0·129 |
|  |  |  | Significance (two-sided) | 0·167 | 0·733 | 0·071 | 0·522 |
|  |  |  | N | 29 | 31 | 30 | 27 |
|  |  | **tc_granulocytes** | Correlationscoeffizient | -0·257 | **-0·549**** | -0·064 | 0·133 |
|  |  |  | Significance (two-sided) | 0·178 | 0·001 | 0·735 | 0·507 |
|  |  |  | N | 29 | 31 | 30 | 27 |
|  |  | **tc_monocytes** | Correlationscoeffizient | -0·168 | -0·223 | 0·010 | -0·201 |
|  |  |  | Significance (two-sided) | 0·384 | 0·228 | 0·957 | 0·314 |
|  |  |  | N | 29 | 31 | 30 | 27 |
|  |  | **tc_cd56** | Correlationscoeffizient | -0·034 | 0·046 | -0·190 | **-0·473*** |
|  |  |  | Significance (two-sided) | 0·859 | 0·805 | 0·315 | 0·013 |
|  |  |  | N | 29 | 31 | 30 | 27 |
|  |  | **tc_cd8_cd58** | Correlationscoeffizient | -0·123 | 0·090 | 0·038 | -0·232 |
|  |  |  | Significance (two-sided) | 0·525 | 0·631 | 0·841 | 0·245 |
|  |  |  | N | 29 | 31 | 30 | 27 |
|  |  | **tc_all_cd45** | Correlationscoeffizient | -0·316 | **-0·534**** | 0·106 | 0·151 |
|  |  |  | Significance (two-sided) | 0·095 | 0·002 | 0·577 | 0·452 |
|  |  |  | N | 29 | 31 | 30 | 27 |
| *. The correlation is significant at the level of 0·05 (two-sided). | | | | | | | |
| **. The correlation is significant at the level of 0·01 (two-sided). | | | | | | | |
|  |  |  |  |  |  |  |  |
|  |  |  | 1 | -1 |  |  |  |

Abbreviations: pO_2_: oxygen partial pressure; DLCO: Diffusion capacity of carbon monoxide; FAS: Fatigue-Assessment-Scale; SMW: Six-minute walking test; tc: Trucount; N: number of patient samples

# **Supplementary Table 5 Average level of antibodies at follow-up**

|  | **All** | **Non-hospitalized** | **Hospitalized non-ICU** | **Hospitalized ICU** | **p-value** |
| --- | --- | --- | --- | --- | --- |
| **IgG S1 (MFI m ± SD) n=123** | 8953·26 (±6170·86) | 5175·54 (± 5225·75) | 12373·16 (±5437·25) | 11884·79 (±4737·98) | **<0·001** |
| **IgG S2 (MFI m ± SD) n=123** | 17542·33 (±6236·51) | 14112·33 (±5835·92) | 20455·34 (±4833·28) | 20369·46 (±5241·14) | **<0·001** |
| **IgG RBD (MFI m ± SD) n=123** | 13543·31 (±6603·74) | 9153·40 (±5693·58) | 17054·26 (±5368·82) | 17348·75 (±4440·18) | **<0·001** |
| **IgG N (MFI m ± SD) n=123** | 15119·79 (±6525·77) | 11855·56 (±6275·83) | 17795·39 (±5335·76 ) | 17893·49 (±5553·76) | **<0·001** |
| **IgM S1 (MFI m ± SD) n=36** | 1249·63 (±1223·99) | 491·68 (±466·12) | 1563·78 (±1228·57) | 1594·00 (±1392·50) |  |
| **IgM S2 (MFI m ± SD) n=36** | 1829·93 (±3045·50) | 726·64 (±729·49) | 1600·17 (±1151·08) | 2717·69 (±4337·67) |  |
| **IgM RBD (MFI m ± SD) n=36** | 3523·16 (±2819·07) | 1900·27 (±1495·18) | 3922·56 (±2362·44) | 4414·25 (±3346·98) |  |
| **IgM N (MFI m ± SD) n=36** | 2156·10 (±2991·08) | 692·64 (±890·97) | 3616·17 (±3720·60) | 2340·94 (±3174·45) |  |
| **IgA S1 (MFI m ± SD) n=36** | 380·42 (334·84) | 232·95 (±222·51) | 466·06 (±372·54) | 433·63 (±362·23) |  |
| **IgA S2 (MFI m ± SD) n=36** | 2285·76 (2293·86) | 1727·55 (±2469·33) | 1785·33 (±1202·55) | 2951·03 (±2567·17) |  |
| **IgA RBD (MFI m ± SD) n=36** | 1231·19 (1018·21) | 989·09 (±1147·80) | 1326·44 (±681·04) | 1344·06 (±1109·69) |  |
| **IgA N (MFI m ± SD) n=36** | 1245·82 (1275·06) | 1258·18 (±1645·54) | 960·06 (±832·72) | 1398·06 (±1242·83) |  |

Abbreviations: IgG/A/M: immunoglobulin G/A/M; S1/S2: Spike-protein S1 / S2 domains; N: Nucleocapsid protein; RBD: Receptor-binding domain

# **Characteristics of acute COVID-19 disease**

Hospitalized and non-hospitalized patients differed significantly in distribution of sex and age. Hospitalized patients were older and were significantly more often male. Hospitalized patients had more risk factors for severe COVID-19 including hypertension (9·0% in non-hos vs 32·0% in non-ICU and 43·8% in ICU; p<0·001), obesity (defined as BMI ≥ 30kg/m^2^: 12·8% in non-hos, 26·0% in non-ICU and 39·6% in ICU; p=0·003), cardiovascular diseases (2·6% in non-hos vs 20·0% in non-ICU vs 22·9% in ICU; p=0.001) and diabetes (1·3% in non-hos vs 12·0% in non-ICU vs 29·2% in ICU; p<0·001). COPD rates did not differ between non-hospitalized and hospitalized non-ICU patients, but where higher in ICU-patients (6·4% and 6·0% vs 20·8%; p=0·017). Age, sex and risk factors did not differ between non-ICU and ICU patients.

During acute disease, at the time of COVID-19 onset, dyspnea was more frequently reported by hospitalized patients (80·0% in non-ICU and 87·5% in ICU vs 52·6% in non-hos; p<0·001), whereas loss of smell, headache and fatigue occurred predominantly in non-hospitalized patients with mild disease (see Table 1). Consistently, WHO Severity Scale was higher in hospitalized patients at disease onset (4 (IQR 3-6) vs. 2 (IQR 2); p<0·001) but did not differ at follow-up visit (2 vs 2; p=0·868). Patients were hospitalized 7 days after symptom-onset at the median, 30 patients (61·2% of hospitalized patients) needed mechanical ventilation, and 8 patients (16·3%) extra-corporeal membrane oxygenation (ECMO).

# **Limitations in dialy life - Questions compiled by the first authors**

**How limited do you feel in daily life since your Covid-19 due to symptoms, pain or psychological stress?**

1. Not at all, I can fulfill all tasks of dialy life without constraints after Covid-19.
2. Slightly, however I still have symptoms, pain or feel psychological stress, I can fulfill all tasks of dialy life.
3. Moderate, I suffer from the consequences of the Covid-19 and avoid or reduce the tasks of dialy life or need more time to fulfill all tasks.
4. Severe, I can not fulfill all tasks of dialy life after the Covid-19, however I am not depend on outside help in dialy life
5. Very strong, I can not fulfill all tasks of dialy life and I am depend on help of another person.

**How do you evaluate the present situation compared to your condition before Covid-19?**

1. Concerning physical performance

| < worse |  | unchanged |  | better > |
| --- | --- | --- | --- | --- |
| ⃝ [1] | ⃝ [2] | ⃝ [3] | ⃝ [4] | ⃝ [5] |

1. Psychological stress (anxiety, worries, depression, nervousness, tension)

| < worse |  | unchanged |  | better > |
| --- | --- | --- | --- | --- |
| ⃝ [1] | ⃝ [2] | ⃝ [3] | ⃝ [4] | ⃝ [5] |

1. Limitations due to symptoms of Covid-19 (e. g. pain, cough, dyspnea)

| < worse |  | unchanged |  | better > |
| --- | --- | --- | --- | --- |
| ⃝ [1] | ⃝ [2] | ⃝ [3] | ⃝ [4] | ⃝ [5] |

# **Details of Patient-reported Outcome Measures**

Quality of life (QoL) was assessed using a visual analogue scale (VAS) ranging from zero points (worst imaginable health state) to ten points (best imaginable health state). Levels of depression and anxiety were assessed using the Hospital Anxiety and Depression Scale (HADS; at MHH) or the Patient Health Questionnaire-9 (PHQ-9) and the Generalized Anxiety Disorder-7 Scale (GAD-7) (at UKE). [1, 2, 3, 4] The HADS questionnaire is divided into subscales for anxiety (HADS-A) and depression (HADS-D), each containing seven questions for a maximum of 21 points per subscale. A cut-off score of eight or above for both HADS-A and HADS-D was used, indicative of clinically relevant levels of anxiety or depression [5]. The PHQ-9 contains nine and the GAD-7 consists of seven items to be rated on a four-point Likert scale (zero = not at all to three = nearly every day). In both scales, all scores are summed up into a total score, with higher scores representing higher levels of depressive and anxiety symptoms, respectively. For both scales a cutoff of ten was used as indicator for clinically relevant symptoms. Anxiety and depression-categories were created using these respective cutoffs. To assess levels of pain patients were asked to state the pain level on a VAS ranging from zero (no pain at all) to ten (worst imaginable pain). Work productivity within the last seven days prior to the study visit was assessed using question five of the Work Productivity and Impairment Index (WPAI-GH) [6] This question covers general work productivity/impairment from zero (current health has no effect on work) to ten (severe effect on work). Patients were asked to omit this question in case they were unemployed.

The © FAS Fatigue Assessment Scale (ild care foundation [www.ildcare.nl)](http://www.ildcare.nl)) consists of ten items with a total sum score of 50 points, 10-21 points indicate no fatigue, whereas ≥ 22 points assume substantial and ≥ 35 points extreme fatigue.

1. Löwe B, Spitzer RL, Gräfe K, Kroenke K, Quenter A, Zipfel S, Buchholz C, Witte S, Herzog W (2004) Comparative validity of three screening questionnaires for DSM-IV depressive disorders and physicians’ diagnoses. J Affect Disord 78:131–140. https://doi.org/10.1016/s0165-0327(02)00237-9

2. Spitzer RL, Kroenke K, Williams JB (1999) Validation and utility of a self-report version of PRIME-MD: the PHQ primary care study. Primary Care Evaluation of Mental Disorders. Patient Health Questionnaire. JAMA 282:1737–1744. https://doi.org/10.1001/jama.282.18.1737

3. Löwe B, Decker O, Müller S, Brähler E, Schellberg D, Herzog W, Herzberg PY (2008) Validation and Standardization of the Generalized Anxiety Disorder Screener (GAD-7) in the General Population. Medical Care 46:266–274. https://doi.org/10.1097/MLR.0b013e318160d093

4. Spitzer RL, Kroenke K, Williams JBW, Löwe B (2006) A Brief Measure for Assessing Generalized Anxiety Disorder: The GAD-7. Arch Intern Med 166:1092. https://doi.org/10.1001/archinte.166.10.1092

5. Bjelland I, Dahl AA, Haug TT, Neckelmann D (2002) The validity of the Hospital Anxiety and Depression Scale. An updated literature review. J Psychosom Res 52:69–77. https://doi.org/10.1016/s0022-3999(01)00296-3

6. Reilly MC, Zbrozek AS, Dukes EM (1993) The validity and reproducibility of a work productivity and activity impairment instrument. Pharmacoeconomics 4:353–365. https://doi.org/10.2165/00019053-199304050-00006
