# Supplementary material for: Blood T cell phenotypes correlate with fatigue severity in post-acute sequelae of COVID-19
Source: Infection. 2023 Nov 4;52(2):513–24. doi: 10.1007/s15010-023-02114-8 (PMC10954951; doi:10.1007/s15010-023-02114-8)
Supplement: Supplementary file 4 — Supplementary file4 (PDF 218 KB) [file 15010_2023_2114_MOESM4_ESM.pdf]

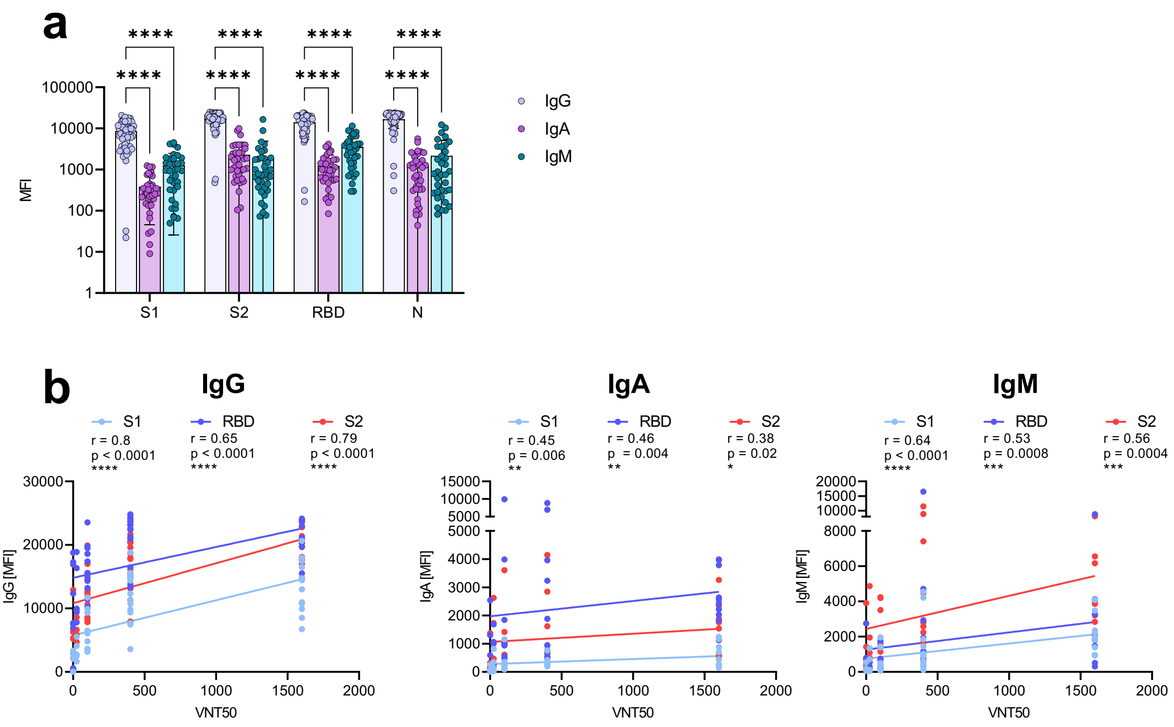

Supplementary Figure 4 Pseudovirus neutralization activity

(a) Luminex-based multiplex assays were used to measure IgG antibody levels against SARS-CoV-2 S1-, S2-domain, RBD and N-antigen in convalescent COVID-19 patients (IgG: n=51, IgA: n=36). **(b)** Correlation analysis between pseudovirus neutralisation titre (VNT50) and IgG, IgA and IgM antibody levels (IgG: n=51, IgA: n=36). Statistical analysis: Two-way repeated measures were performed using ANOVA test with Tukey multiple comparisons test, correlation analysis were performed using Spearman correlation. *p < 0·05, **p < 0·01, ***p < 0·001, ****p < 0·0001
